# Phosphofructokinases A and B from *Mycobacterium tuberculosis* Display Different Catalytic Properties and Allosteric Regulation

**DOI:** 10.3390/ijms22031483

**Published:** 2021-02-02

**Authors:** Jan Snášel, Iva Machová, Veronika Šolínová, Václav Kašička, Marcela Krečmerová, Iva Pichová

**Affiliations:** Institute of Organic Chemistry and Biochemistry of the Czech Academy of Sciences, Flemingovo nám. 2, 16610 Prague 6, Czech Republic; jan.snasel@uochb.cas.cz (J.S.); Iwik.m@seznam.cz (I.M.); veronika.solinova@uochb.cas.cz (V.Š.); vaclav.kasicka@uochb.cas.cz (V.K.); marcela.krecmerova@uochb.cas.cz (M.K.)

**Keywords:** *Mycobacterium tuberculosis*, glycolysis, phosphofructokinase A and B, allosteric regulation, enzyme kinetics

## Abstract

Tuberculosis (TB) remains one of the major health concerns worldwide. *Mycobacterium tuberculosis* (Mtb), the causative agent of TB, can flexibly change its metabolic processes during different life stages. Regulation of key metabolic enzyme activities by intracellular conditions, allosteric inhibition or feedback control can effectively contribute to Mtb survival under different conditions. Phosphofructokinase (Pfk) is one of the key enzymes regulating glycolysis. Mtb encodes two Pfk isoenzymes, Pfk A/Rv3010c and Pfk B/Rv2029c, which are differently expressed upon transition to the hypoxia-induced non-replicating state of the bacteria. While *pfkB* gene and protein expression are upregulated under hypoxic conditions, Pfk A levels decrease. Here, we present biochemical characterization of both Pfk isoenzymes, revealing that Pfk A and Pfk B display different kinetic properties. Although the glycolytic activity of Pfk A is higher than that of Pfk B, it is markedly inhibited by an excess of both substrates (fructose-6-phosphate and ATP), reaction products (fructose-1,6-bisphosphate and ADP) and common metabolic allosteric regulators. In contrast, synthesis of fructose-1,6-bisphosphatase catalyzed by Pfk B is not regulated by higher levels of substrates, and metabolites. Importantly, we found that only Pfk B can catalyze the reverse gluconeogenic reaction. Pfk B thus can support glycolysis under conditions inhibiting Pfk A function.

## 1. Introduction

*Mycobacterium tuberculosis* (Mtb) is an infectious agent that causes human tuberculosis (TB), a disease among the top 10 causes of death worldwide. Approximately 10 million people develop TB annually and 23% of the world’s population is estimated to have latent Mtb infection with a risk for developing active TB. Treatment is complicated by the spread of multidrug resistant Mtb strains (WHO report for 2020 [1]). Furthermore, TB is the most common opportunistic infection among HIV-positive patients and co-infection affects the disease progression [2]. Other factors such as diabetes mellitus, patient age and other social determinants of health also contribute to high TB incidence [3,4]. Current understanding of Mtb replication and pathogenesis indicates that these bacteria have a highly flexible metabolism adaptable to diverse physiological stages. Mtb can co-catabolize multiple carbon sources by different metabolic pathways occurring in a segregated manner, and this allows the bacteria to enter into the non-replicative state of dormancy [5]. The available data show that metabolic enzymes with specific properties and carefully regulated activities contribute substantially to Mtb metabolic changes (for review, see [6]).

Glycolysis, a pathway at the center of carbon metabolism that generates fundamental metabolites including phosphoenolpyruvate (PEP), pyruvate and acetyl-CoA, is highly conserved in all living organisms. In growing Mtb, glucose is primarily oxidized through glycolysis, and only a small fraction enters the pentose phosphate cycle [7]. The reaction catalyzed by phosphofructokinase (Pfk) is one of the most important rate-limiting steps in glycolysis. In most organisms, Pfk functions in a single direction, catalyzing the production of fructose-1,6-bisphosphate (F16bP) from fructose-6-phosphate (F6P) and ATP. The Mtb genome encodes two ATP-dependent 6-phosphofructokinases: Pfk A (Rv3010c) and Pfk B (Rv2029c). The *pfkA* and *pfkB* genes are differently localized. Pfk B is a member of the *devR/DosR* regulon [8] and its gene and protein expression are upregulated during hypoxia, while Pfk A is down-regulated during growth arrest [9,10]. Although both enzymes are thought to catalyze the same enzymatic reaction, they belong to different subfamilies. Pfk A belongs to the Pfk protein family, whereas Pfk B belongs to the Pfk B subfamily of ribokinases [11]. Bacterial Pfk enzymes are usually tetrameric and allosterically activated by different metabolites [12]. In most organisms, the opposite reaction, hydrolysis of F16bP to F6P, an important rate-limiting step in gluconeogenesis, is catalyzed by fructose-1,6-bisphosphatase (FBPase, *glpX*) [13]. In Mtb, an additional broad-specificity fructose bisphosphatase, GPM2 (Rv3214), that catalyzes the same reaction, has been identified. Both Mtb FBPase and GPM2 appear to be involved in gluconeogenesis, as deletion of both genes is required to disrupt the pathway [13]. Pfks from several microorganisms can also catalyze the opposite reaction to some extent, and thus partially supplement or substitute for the activity of FBPase [14,15]. Metabolic flow through gluconeogenesis is necessary for Mtb infection and persistence in macrophages [16,17] and is indispensable for converting tricarboxylic acid cycle intermediates into sugars [18] when Mtb uses fatty acids as a carbon source. According to Phong et al. [19], Pfk A is the main glycolytic enzyme in Mtb.

Here, we compared the enzymatic properties of both Pfk isoenzymes and showed that both can catalyze biosynthesis of F16bP although with different efficiency. In addition, we found that only Mtb Pfk B is able to catalyze the reverse gluconeogenic reaction. Further, we showed that Pfk A activity is significantly more sensitive to the feedback effect of F16bP, and is allosterically inhibited by phosphoenolpyruvate (PEP), guanosine diphosphate (GDP), and citrate. Pfk B is not inhibited by an excess of substrates and common allosteric inhibitors. These data suggest that the role of Pfk B is to support and maintain basic glycolysis and metabolic fluxes during conditions inhibiting Pfk A.

## 2. Results

### 2.1. The Kinetics of Glycolytic Reactions Catalyzed by Pfk A and Pfk B Differ Significantly

First, we established expression and purification procedures for both Pfk isoenzymes. We produced Pfk A and B in *E. coli* BL21(DE3) cells containing the pGKJE8 plasmid, which expresses chaperone proteins that facilitate solubility of recombinant enzymes. Since Pfk A and B share low percentages of amino acid sequence identity (14%) and similarity (20%) (see Figure S1A), we employed slightly different purification procedures for each enzyme (see Materials and Methods). The N-terminal amino acid sequencing of bands corresponding to about 37 kDa (Figure S2A,B) confirmed presence of sole Pfk A and Pfk B. These enzyme fractions were used for characterization.

**Figure 1:**
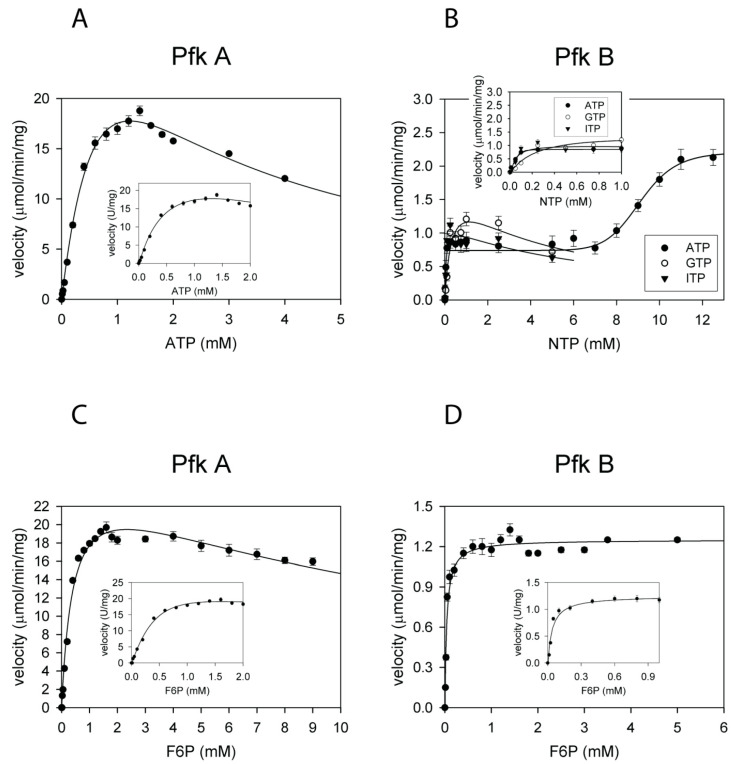
Influence of nucleoside triphosphate (NTP) concentration on glycolytic activities of phosphofructokinase (Pfk) A and Pfk B. Dependencies of Pfk A (**A**) and Pfk B (**B**) reaction velocities on NTP concentration. The concentration of F6P was kept at 1 mM while the concentration of NTPs varied between 0–12 mM. Error bars are standard deviations. Influence of F6P concentration on glycolytic activities of Pfk A and Pfk B. Dependencies of Pfk A (**C**) and Pfk B (**D**) activities on increasing fructose-6-phosphate (F6P) concentrations. The concentration of F6P varied between 0–10 or 0–6 mM while the concentration of ATP was fixed at 1 mM. Error bars are standard deviations. The data in panel (**A**) were fitted to Equation (4), in panel (**B**) to Equations (3) and (4), in panel (**C**) to Equation (4), and in panel (**D**) to Equation (1).

The glycolytic activities (synthesis of F16bP) of Pfk A and B were determined spectrophotometrically by monitoring the decrease in absorbance at 340 nm during NADH oxidation to NAD^+^ in coupled reactions with aldolase, α-glycerophosphate dehydrogenase, and triosephosphate isomerase (coupled assay I). The reaction velocities were monitored at a fixed concentration of one substrate (1 mM F6P or ATP) and varied concentration of the second substrate (0–12 mM ATP or 0–50 mM F6P). The initial velocity data were fitted to either the Michaelis-Menten equation (Equation (1)), sigmoidal (Hill) response curve (Equation (2)), biphasic response curves (Equation (3)), or Haldane’s equation for substrate inhibition (Equation (4)). The dependence of Pfk A activity on the concentration of ATP followed sigmoidal kinetics (Figure 1A). However, the enzyme was gradually inhibited by an excess of ATP (>1.5 mM). Other nucleoside triphosphates (GTP, ITP) were not accepted as phosphate donors in the Pfk A mediated reaction. Interestingly, the dependence of Pfk B reaction velocity on the concentration of ATP indicated biphasic kinetics (Figure 1B and Table 1). The velocity increased proportionally with increasing ATP concentration up to 0.2 mM and reached a relatively long plateau region at concentrations between 0.2 and 6 mM. Further increase in ATP concentration led to a faster reaction rate until the maximum reaction velocity was attained (2 µmol/min/mg) GTP and ITP were used as phosphate donors only in the Pfk B-mediated reaction but with typical Michaelis-Menten hyperbolic kinetics. The GTP or ITP concentrations higher than 1 mM gradually inhibited Pfk B (Figure 1B).

The dependence of Pfk A activity on F6P concentration was sigmoidal up to 2 mM F6P. Higher F6P concentrations slightly inhibited Pfk A (Figure 1C). The dependence of Pfk B activity on F6P concentration showed typical Michaelis-Menten hyperbolic kinetics (Figure 1D and Table 1), and higher concentrations of F6P did not inhibit the enzyme. Our kinetics analyses indicated significant differences in inhibition of Pfk A and B by increasing ATP concentrations. While higher concentrations of ATP inhibited Pfk A, the Pfk B isoenzyme catalyzed the glycolytic reaction at increased ATP concentrations without any significant change in efficiency.

### 2.2. Pfk B, but Not Pfk A, Detectably Catalyzes the Gluconeogenic Reaction

**Figure 2:**
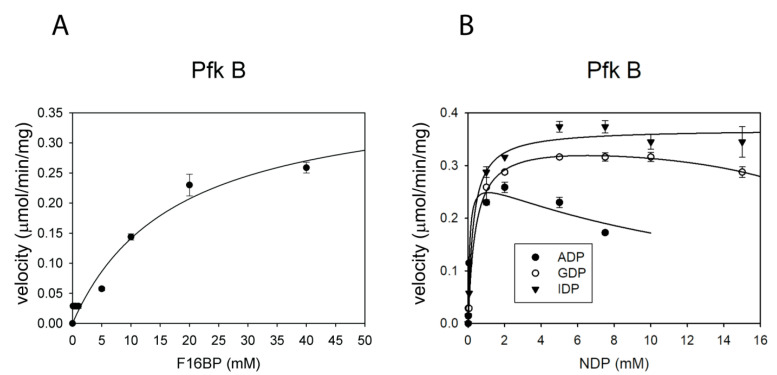
Gluconeogenic activity of Pfk B. (**A**) Dependence of Pfk B activity on the concentration of fructose-1,6-bisphosphate (F16bP). The concentration of ADP was kept at 1 mM while the concentration of F16bP was varied between 0–50 mM. (**B**) Dependence of Pfk B activity on the concentration of nucleoside diphosphates (NDPs). The concentration of F16bP was kept at 5 mM while the concentration of NDP was varied between 0–20 mM. Error bars are standard deviations from three independent experiments. The data in panel (**A**,**B**) were fitted to Equations (1) and (4).

To determine whether Mtb Pfk A or Pfk B can catalyze detectably the reverse reaction, we measured the activity of both enzymes in the presence of a fixed concentration of ADP (1 mM) and varying concentrations of F16bP (0–50 mM), and in the presence of 5 mM F16bP with varying concentrations of ADP (0–20 mM). As low reaction yields were expected, we monitored activity using coupled enzyme reactions with phosphoglucose isomerase and glucose-6-phosphate dehydrogenase. Pfk A did not display activity under any of the conditions tested, but Pfk B catalyzed the gluconeogenic reaction, albeit with low efficiency (Figure 2A,B; Table 2). Comparing the kinetic parameters of the Pfk B-catalyzed glycolytic and gluconeogenic reactions revealed that the low catalytic efficiency of the gluconeogenic reaction is largely due to the low binding affinity of F16bP to Pfk B, reflected in a relatively high K_m_ value (17.5 mM). Interestingly, GDP and inosine diphosphate (IDP) can also serve as acceptors of the phosphate group in the Pfk B-catalyzed gluconeogenic reaction (Figure 2B; Table 2). These results suggest that Pfk B might, in addition to fructose-1,6-bisphosphatase (*glpX*), support the biosynthesis of F6P under certain metabolic conditions in Mtb.

### 2.3. Magnesium Is Essential for Pfk A and B Catalysis but Affects Their Activities with Different Mechanism

**Figure 3:**
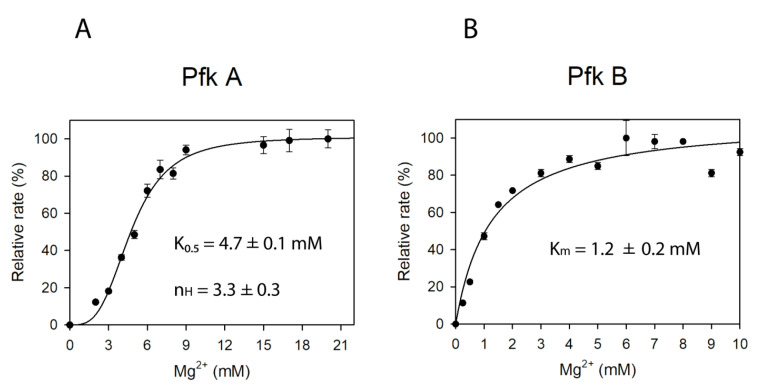
The dependence of Pfk A and Pfk B activities on concentration of Mg^2+^. Individual reactions were performed in buffers containing 1 mM F6P and 1 mM ATP with the concentration of Mg^2+^ varied. The data were fitted to Equation (2) (**A**) and Equation (1) (**B**). The velocities were normalized (100% determines the maximum achievable velocity).

The activities of both Pfks were found to be proportionally dependent on Mg^2+^ concentration. Accordingly, no activities were detected with both Pfks in the absence of Mg^2+^. As shown in Figure 3A Pfk A displayed a cooperative sigmoidal kinetic response to increasing Mg^2+^ concentrations with a Hill coefficient of 3.3, suggesting a role of this cation in formation and maintaining of proper enzyme conformation and multimerization. On the contrary, the Pfk B activity dependence on Mg^2+^ concentration was hyperbolic (Figure 3B), which indicates that magnesium does not function as an allosteric effector of Pfk B but is essential for glycolytic reaction.

To further analyze the allosteric effect of magnesium ions on Pfk A, the activity dependencies were measured at fixed concentrations of one substrate (F6P, ATP) and varying concentrations of the second substrate (0–20 mM F6P or ATP) (Figure S3). When the ATP concentration was fixed and F6P varied, the increased Mg^2+^ concentration decreased the Hill coefficient for F6P. On the other hand, when F6P was fixed and ATP varied, the Hill coefficient for ATP was increased. Altogether, these results show that F6P and ATP substrates have different effects on resulting Pfk A multimeric state with respect to the Mg^2+^ concentration.

### 2.4. Pfk A, but Not Pfk B Activity Is Allosterically Regulated by Metabolites

Characterization of Pfks from different sources has shown that their glycolytic activities can be regulated by allosteric effectors that bind to multiple sites within the multimeric structures of these enzymes [12,20]. We, therefore, investigated the possible allosteric regulatory effects of PEP, GDP, AMP, G6P, R5P, metabolites from closely related pathways, on Pfk A and B enzymatic activities.

**Figure 4:**
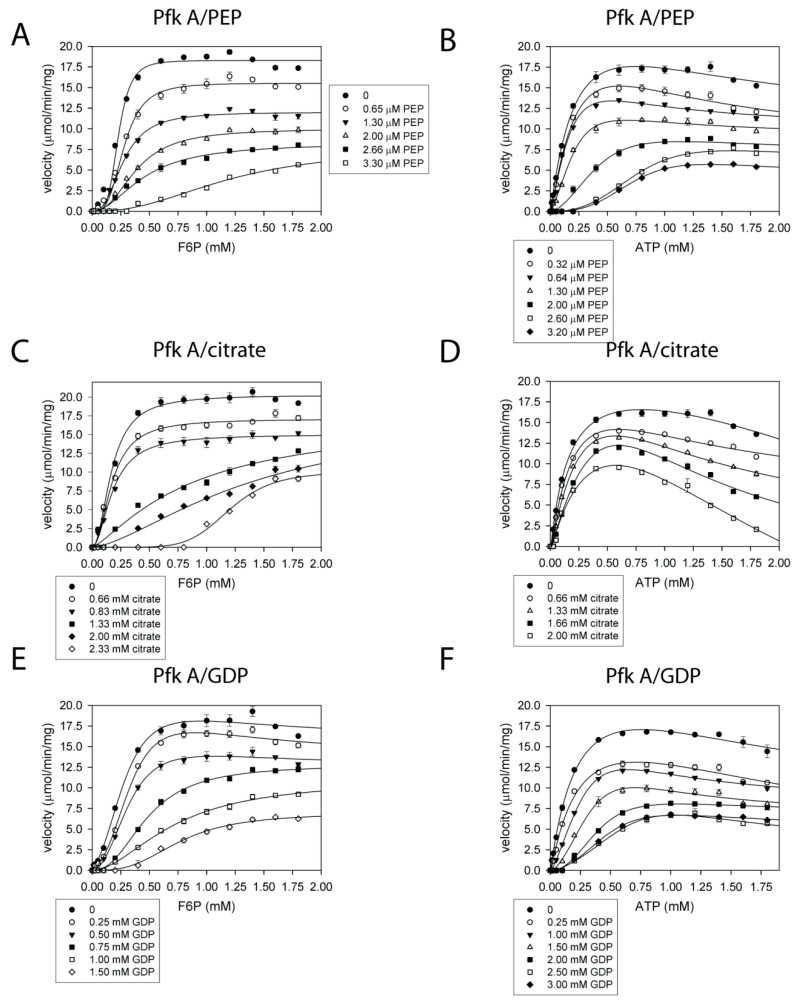
The inhibition of Pfk A by PEP (**A**,**B**), citrate (**C**,**D**), and GDP (**E**,**F**). Plots of the inhibitions with F6P varied and ATP fixed at 1 mM (left diagrams: **A**,**C**,**E**) and the inhibitions with ATP varied and F6P fixed at 1 mM (right diagrams: **B**,**D**,**F**). The data were fitted to Equations (2) and (4).

We observed some effect on Pfk A only for PEP, citrate, and GDP. The PEP inhibition patterns (Figure 4A,B) measured either at fixed concentration of ATP (1 mM) and varying F6P concentrations (0–2 mM) (Figure 4A) or in the presence of 1 mM F6P and ATP concentrations ranging from 0–2 mM (Figure 4B) revealed that the efficient Pfk A inhibition can be achieved by a low micromolar concentration of this metabolite. Citrate also inhibited Pfk A, however, in millimolar concentrations (Figure 4C,D). Pfk A was found to be efficiently inhibited also by GDP within the millimolar concentration range (Figure 4E,F). The patterns of double reciprocal plots for PEP (Figure S4A,B), citrate (Figure S4C,D), and GDP (Figure S4E,F) as well as increased Hill coefficients in the presence of higher effector concentrations revealed the allosteric mode of inhibitions by all these metabolites. Similarly, both types of Dixon plots (data not shown) in the presence of varying concentrations of F6P or ATP indicated multisite inhibition of Pfk A by these metabolites: these data suggest that binding of one molecule of allosteric inhibitor to the enzyme multimer induces structural changes that facilitate binding of another inhibiting molecule.

In contrast to Pfk A, the Pfk B activity was marginally inhibited only by a non-physiologically PEP concentration higher than 5 mM (data not shown).

To check if the observed effects of PEP, GDP, and citrate on Pfk A activity are not due to inhibition of enzymes involved in coupled enzyme assay (i.e., triosephosphate isomerase, aldolase, glycerol-3-phosphate dehydrogenase), we ran the assay without Pfk A in the presence of 0.3 mM NADH, 1 mM F16bP and increasing concentrations of metabolites. Figure S5 indicates that PEP did not inhibit coupled enzymes until PEP concentrations reached 5 µM while significant inhibition of the enzyme activity was observed in the system with Pfk A and 2–3 µM PEP. Citrate and GDP did not inhibit the coupled enzyme system without Pfk A at concentrations up to 1.5 mM and 3 mM, respectively. These data show that PEP, citrate, and GDP directly affect Mtb Pfk A at conditions tested.

### 2.5. Effect of Fructose-2,6-Bisphosphate on Pfk Isoenzymes

In general, fructose 2,6-bisphosphate (F26bP) is considered as a potent activator of most eukaryotic phosphofructokinases. F26bP activates Pfks by increasing their affinity for F6P and diminishing the inhibitory effect of ATP [21,22]. We synthetized F26bP and tested its influence on Pfk A and Pfk B catalyzed reactions under different conditions. The effect of increasing concentrations of F26bP on the Pfk A and Pfk B activities in the presence of 1 mM F6P and ATP illustrates Figure S6. Only higher concentrations of F26bP than 50 µM slightly activated both Pfks. Presence of 100 µM F26bP activated Pfk A and Pfk B by 10 and 20%, respectively. Similar trends were observed at varied F6P concentration (0–1 mM) and fixed ATP concentration (1 mM) and vice versa (F6P concentration fixed at 1 mM, ATP concentration varied between 0–4 mM) (data not shown). No effect of F26bP (50 µM) on Pfk A inhibition by 1 mM citrate or 1 µM PEP or ATP excess (up to 10 mM, data not shown) was detected. Our experiments indicated that Pfk B activity is more sensitive to higher concentrations of F26bP.

### 2.6. Pfk A and Pfk B Activities Are Controlled by Negative Feedback by Both Reaction Products but with Different Efficiency

Further, we tested whether Pfk A and Pfk B can be either activated or inhibited by both reaction products (F16bP, ADP). Since F16bP is a substrate of aldolase involved in the coupled enzyme system I, we used coupled assay system II with pyruvate kinase and lactate dehydrogenase for feedback analysis of this reaction product on Pfk glycolytic activity. However, we had to modify this system, since we showed that PEP, the second substrate of pyruvate kinase in the reaction system II, inhibits the Pfk A glycolytic activity. In this modified system, the Pfk reaction was firstly run as an uncoupled reaction for 5 min followed by the PEP addition, which initiated subsequent pyruvate kinase and lactate dehydrogenase coupled reactions. The reaction velocity was directly proportional to the formed ADP and corresponded to the average velocity during the time interval of 5 min (for details see Materials and Methods, Section 4.7). The reactions were measured in the presence of different concentrations of both products and fixed concentration of one substrate (1 mM) and varied concentrations of the second substrate (0–2 mM). The effect of ADP was monitored using coupled enzyme assay I.

**Figure 5:**
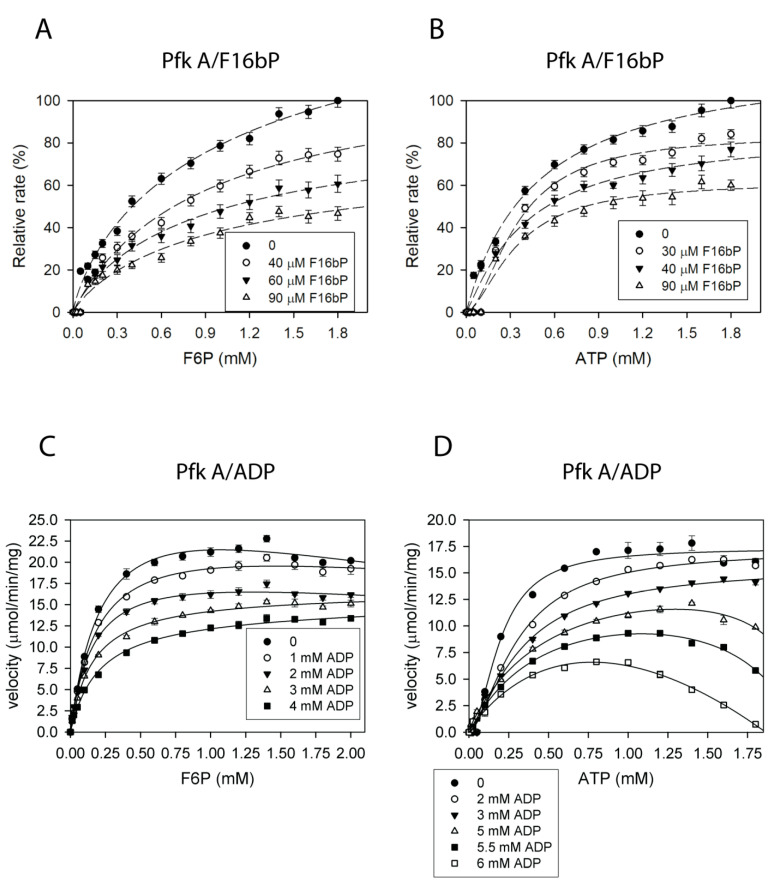
The feedback inhibition of Pfk A by F16bP (**A**,**B**) and ADP (**C**,**D**) measured at 1 mM ATP (**A**,**C**) or F6P (**B**,**D**) and varied concentration of a second substrate. Individual reaction mixtures of the coupled enzyme system II (see Materials and Methods) containing appropriate concentrations of F6P and ATP in the absence of phosphoenolpyruvate (PEP) were incubated with 20 nM Pfk A for 5 min at 30 °C. Thereafter, PEP was added to the 1 mM final concentration and the progress of the reaction was followed by measuring the absorbance at 340 nm. The average reaction velocities corresponding to the ratios of the formed ADP and starting ATP concentration in 5 min were plotted in graphs (**A**,**B**). For better illustration the velocities were normalized (100% determines the maximum achievable velocity). The dashed lines indicate the increasing trend. The reactions were run according to Materials and Methods using coupled enzyme assay I (**C**,**D**). The data were fitted to Equations (2) and (4).

Our results showed that Pfk A was efficiently inhibited by F16bP within the low micromolar concentration range (0–100 µM) (Figure 5A,B). ADP also inhibited Pfk A (Figure 5C,D) but in low millimolar concentrations (2–5 mM). Interestingly, as seen in Figure 5D, the ADP concentrations higher than >5 mM gradually elicited also inhibition by ATP.

**Figure 6:**
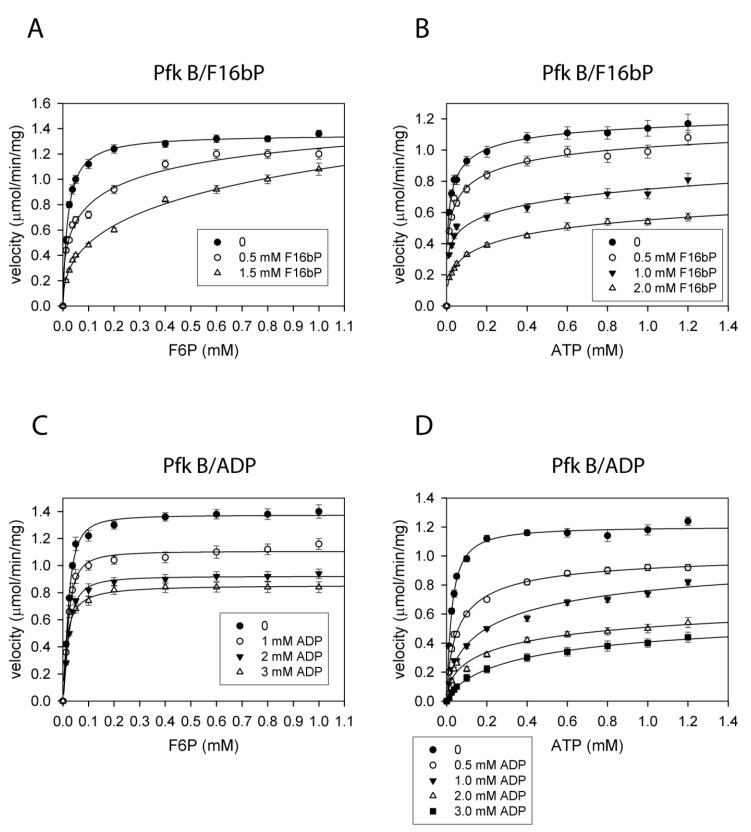
The feedback inhibition of Pfk B by F16bP (**A**,**B**) and ADP (**C**,**D**). Plots of the inhibitions with F6P varied and ATP fixed at 1 mM (**A**,**C**) and the inhibitions with ATP varied and F6P fixed at 1 mM (**B**,**D**). The data were fitted to Equation (1).

Since Pfk B was not substantially inhibited by PEP, the unmodified coupled reaction system II was used to analyze a feedback effect of F16bP on glycolytic activity. Both reaction products F16bP (Figure 6A,B) and ADP (and Figure 6C,D) inhibited Pfk B in mM ranges. Taken together, the results indicate that Pfk A is more sensitive to the feedback control by F16bP.

### 2.7. Pfk A and B Can Convert Fructose-6-Phosphate and Tagatose-6-Phosphate

**Figure 7:**
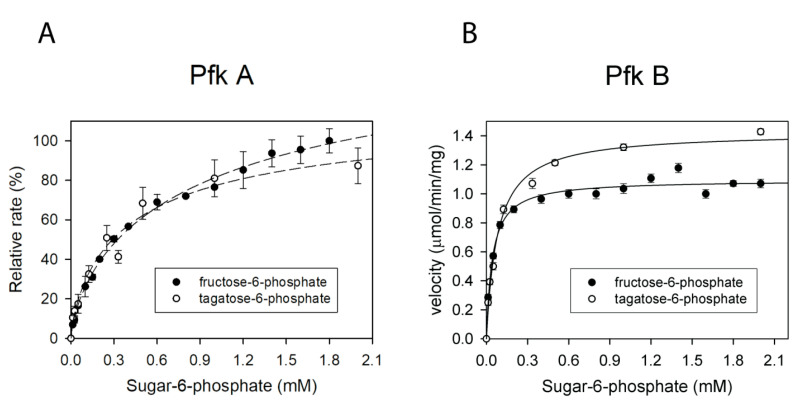
Utilization of different sugar-phosphates as substrates in the Pfk A and Pfk B reaction. (**A**) Coupled reaction mixture II without PEP containing 1 mM ATP and 30 nM Pfk A was aliquoted to individual microtiter plate wells containing increasing concentrations of the particular sugar-phosphate (0–2 mM F6P or T6P). Reactions were allowed to proceed for 5 min and subsequently PEP solution was pipetted into each well to reach 1 mM final concentration. 100% corresponds to the highest reaction rate (enzyme activity) achievable. Dashed lines indicate the increasing trend. (**B**) Coupled reaction mixture II with 1 mM ATP and 300 nM Pfk B was aliquoted to individual microtiter plate wells containing increasing concentrations of the particular sugar-phosphate (0–2 mM F6P or T6P). The data were fitted to Equation (1).

We next investigated substrate specificity of Pfk A and Pfk B using different sugar monophosphates. We tested fructose-1-phosphate (F1P), tagatose-6-phosphate (T6P), sedoheptulose-7-phosphate (S7P), glucose-6-phosphate (G6P) and ribulose-5-phosphate (Rib5P). For Pfk A we used the modified enzyme assay II and for Pfk B the unmodified system II (for details see Materials and Methods, Section 4.4.1). The reactions were measured with fixed ATP concentration (1 mM) and varied concentrations of sugar monophosphates. From the set of tested sugar monophosphates, the detectable Pfk A activity was found only for F6P and T6P. The kinetic parameters K_1/2_ and V_max_ values for F6P and T6P were comparable (Table 3), which clearly indicates that both sugar phosphates can function equivalently as Pfk A substrates (Figure 7A and Figure S7). Similarly, Pfk B catalyzed only phosphorylation of T6P and F6P (Figure 7B). The determined K_1/2_ value for T6P (0.09 mM) was about twice higher than that for F6P (0.04 mM). On the other hand, maximum velocity with T6P (1.5 µmol·min^−1^·mg^−1^) was by 25% higher than that with F6P (1.2 µmol·min^−1^·mg^−1^). The catalytic efficiency of Pfk B with T6P was about 1.8 times lower than that with F6P and about 4 times lower than in the presence of Pfk A (Table 3).

We also used an alternative direct method for analysis of Pfk enzyme specificities and monitored ATP conversion into ADP using capillary electrophoresis (CE). To achieve detectable conversion of ATP, we used 20 nM Pfk A and 200 nM Pfk B enzymes in reactions containing 1 mM ATP and 1 mM sugar phosphates. The reactions were linear for a time period up to 15 min at 30 °C. The results confirmed that both Pfk enzymes effectively accept only F6P and T6P for phosphorylation (Table S1 or Figure S8).

### 2.8. Pfk B Is Not a Member of PP_i_-Pfk Family

Taking into consideration the low glycolytic activity of Pfk B, its ability to catalyze the reverse gluconeogenic reaction, atypically low degree of regulation by the feedback effect as well as no allosteric response to typical allosteric small-molecule effectors, we further checked possibility that Pfk B might be a pyrophosphate-dependent Pfk (PP_i_-Pfk; EC 2.7.1.90) accepting pyrophosphate instead of ATP [23,24]. We tested activity of Pfk B in the presence of a broad concentration range of PP_i_ (50 µM–5 mM) and a fixed concentration of F6P (0.5 mM) (for details see Materials and Methods, Section 4.3). However, no PP_i_ dependent activity of Pfk B was determined thus confirming that Pfk B functions as a pure ATP dependent enzyme (data not shown). Similar results were obtained for Pfk A (data not shown).

## 3. Discussion

The cellular metabolism of Mtb is flexible and can be adapted to various environmental stress conditions, which are accompanied by different metabolite levels and compositions. Mtb can co-catabolize multiple carbon sources, enabling segregated but simultaneous carbon fluxes through multiple metabolic pathways including glycolysis and gluconeogenesis [5]. Control of metabolic enzyme activities by the cellular environment and allosteric effectors contributes to adaptive switches in metabolism [25,26]. Significant metabolic changes occur during Mtb’s transition to a persistent state. Under hypoxic conditions, Mtb replication is slowed, and gluconeogenic flux plays an important role [13]. As the bacterium enters the latent phase, most transcripts encoding key glycolytic and pentose phosphate pathway (PPP) enzymes are downregulated, with the exception of the *pfkB* gene, which is significantly upregulated [9]. Absolute quantification of protein abundance by mass spectrometry analysis confirmed a five-fold increase in Pfk B protein expression upon hypoxia [10]. Overall, the role of Pfk B in Mtb metabolism remains puzzling.

Here, we performed the detailed biochemical characterization of both isolated Mtb Pfks, in order to find out if enzymatic properties of these enzymes might contribute to their function. Our results showed that intracellular conditions, i.e., concentration of enzyme substrates, products, magnesium, or metabolites can play an important role in resulting Pfk A and Pfk B activities. The Pfk A specific velocity gradually decreased at ATP concentrations 1.5 mM and higher. Increasing concentration of ATP may allosterically change the affinity of other binding sites and may result in a non-productive substrate binding mode and enzyme inhibition. Similarly, Fenton and Reinhart [27] demonstrated that Mg-ATP inhibit *E. coli* Pfk in the presence of low concentrations of F6P. Interestingly, Pfk B activity exhibits a biphasic dependence on ATP concentration, suggesting reorganization of enzyme multimers at certain ATP levels. Enzymes exhibiting atypical biphasic kinetics are very rare in nature and the only known example of phosphofructokinases showing such a kinetic profile is sea bass (*Dicentrarchus labrax* L.) phosphofructokinase which produces biphasic kinetics with respect to F6P [28]. The F6P concentrations higher than 1.5 mM also lead to the partial inhibition of Pfk A but had no effect on Pfk B, suggesting the existence of alternative binding sites for F6P within the Pfk A multimer. Only Pfk B can use phosphate donors other than ATP (GTP and ITP) but with lower efficiency and different kinetics. Higher concentrations of GTP and ITP (>1 mM) inhibiting Pfk B, possibly serve as a negative feedback control. Our kinetic data show that mycobacterial Pfk A is about 10 times slower than Pfk 1 from *E. coli* (V_max_ = 190 µmol·min^−1^·mg^−1^) [29]. However, sequence alignment of these two enzymes indicates low identity and similarity (see Figure S1B).

Although Pfk B is less active than Pfk A, its unique features enable catalysis in the gluconeogenic direction, producing F6P from F16bP. The low catalytic efficiency (V_max_/K_m_) of the gluconeogenic reaction is due to the high K_m_ value for F16bP. Such low catalytic efficiency in the gluconeogenic reaction has also been observed for Pfks from *E. coli* and *B. stearothermophillus* [14,15,30]. For the ATP-dependent Pfks, the forward reaction is favored under physiological conditions and the reverse reaction has never been demonstrated for any eukaryotic ATP-dependent Pfks in vitro or in vivo [31]. Recently, the kinetic parameters of the reverse (gluconeogenic) reaction were determined for Pfks from several parasites (Pfk from *Trypanosoma brucei*, V_max_ = 4.2 µmol·min^−1^·mg^−1^; Pfk from *Leishmania* spp., V_max_ = 3.8 µmol·min^−1^·mg^−1^) [31]. These values are 10 times higher than here determined kinetic parameters for the gluconeogenic reaction of Pfk B (V_max_ = 0.4 µmol·min^−1^·mg^−1^). Characterization of other important Mtb metabolic enzymes like pyruvate kinase [26], phospho(enol)pyruvatecarboxy kinase [25] also reported their unique properties, e.g., slow catalysis, different allosteric regulation, influence of reducing conditions on reverse reaction compared to corresponding bacterial or human enzymes. The slower kinetic parameters of Mtb phosphofructokinases correspond well with the slower metabolism of the pathogenic bacteria, which is manifested by extremely slow growth rates. Enzyme properties thus represent additional factor contributing to regulation of metabolic fluxes during different stages of Mtb life cycle.

Pfks from different species can phosphorylate a variety of mono-phosphorylated sugars [29,32,33,34]. We found that both Pfk A and B phosphorylate not only F6P but also T6P, suggesting their potential involvement in biosynthesis of d-glyceraldehyde-3-phosphate and dihydroxyacetone phosphate through conversion from d-tagatose-1,6-bisphosphate by a tagatose-bisphosphate aldolase [35]. Several microorganisms, including *Staphylococcus aureus*, encode tagatose-6-phosphate kinase (*LacC*), a specific enzyme that catalyzes phosphorylation of tagatose-6-phosphate [34]. Interestingly, the tertiary structures of the LacC enzyme and some members of the *pfkB* subfamily of carbohydrate kinases are similar. As a gene encoding a potential tagatose-6-phosphate kinase has not been identified in the Mtb genome, one can speculate that Pfk A or Pfk B can substitute for tagatose-6-phosphate kinase and further support glycolysis.

Importantly, we found allosteric negative regulation of Pfk A activity by PEP and citrate, which inhibits also some other mammalian Pfks [36,37], and *E. coli* Pfk [38]. We also observed negative feedback effect of ADP and F16bP on Pfk A catalysis. Interestingly, we have not detected any activation of Pfk A with metabolites tested here. Neither Pfk A nor Pfk B was significantly activated by F26bP in contrast to Pfk(s) from mammalian tissues, some fungi, *Saccharomyces cerevisiae*, *Aspergillus niger* that are stimulated by this compound [21,39,40,41]. Presence of F26bP did not lead to the reversal Mtb Pfk A inhibition by ATP [21]. Surprisingly, our results showed that although Pfk B is apparently comprised of several subunits, it is not a subject to allosteric control by physiological concentrations of metabolites tested here. The existence of two Mtb Pfk isoforms that differ in sensitivity to allosteric effectors may have a deeper, however not yet fully explained biological significance. The catalytically active form of most bacterial Pfks is a homotetramer that contains four identical active sites and four identical allosteric binding sites. However, the binding affinities of allosteric ligands, as well as the activity responses of various bacterial Pfks, differ [38,42,43,44]. In general, glycolysis can be regulated by three allosterically regulated enzymes. The upper part of glycolysis is controlled by the multi-subunit enzymes hexokinase (glucokinase-Gk) and Pfk, and the lower glycolytic part is controlled by pyruvate kinase (Pyk). To keep the metabolic balance in equilibrium, all catalytic steps should proceed in a concerted fashion with carefully controlled mutual interplay among regulatory enzymes. It is well-established that Pfks contribute to oscillations in the concentration of glycolytic intermediates (G6P, F6P, F16bP) due to regulation of their enzymatic activity by reaction products (F16bP, ADP) and substrates (F6P, ATP). In this model, the reaction speeds up as product concentrations increase, until the substrates are depleted and the reaction ceases [45]. However, we found that F16bP and ADP may act as negative effectors of Pfk A, which suggests that Mtb Pfk A is the only Pfk characterized to date that is not regulated by F16bP positive feedback. As Mtb Pfk A is negatively controlled by an excess of both substrates (F6P and ATP) and products (F16bP and ADP), oscillations in concentrations of glycolytic intermediates are likely triggered and maintained by a mechanism other than mutual interplay between positive and negative feedback control of Pfk. Interestingly, we and others have shown that G6P and F16bP are allosteric activators of Mtb Pyk [26,46]. Such opposite modes of Pfk A and Pyk regulation by common metabolites may form a functional metabolic switch between anaerobic glycolysis and gluconeogenesis.

The conversion of F16bP to F6P in Mtb can be catalyzed by the allosterically regulated enzymes F16BPase (*glpX*) and the broad-specificity phosphatase GPM2 [13]. However, Pfk B might be also a representative of pyrophosphate dependent phosphofructokinases (EC 2.7.1.90) utilizing PP_i_ instead of ATP during glycolysis. PP_i_-Pfk, which were found mainly in anaerobic bacteria and autotrophic bacteria, can catalyze both, the glycolysis and gluconeogenesis [24,47], are not allosterically activated by organic compounds and their activities are rather regulated at the level of substrate and product concentrations. However, we have not detected any Pfk B activity with broader pyrophosphate substrate concentration range at conditions in which enzymes constituting this class perform efficiently [24,48,49]. Our results confirmed that Pfk B belongs to the ATP dependent phosphofructokinases.

In summary, our results indicate that Mtb Pfk A and B have different biochemical properties that are unique among bacterial and other microbial species. Phong et al. [19] provided evidence that Pfk A is the only phosphofructokinase in the Mtb metabolism and Pfk B does not contribute to glycolysis. However, our detailed biochemical characterization revealed that Pfk B is active at least in vitro under a broad spectrum of conditions (pH ranges 6.5–9.0 and ionic strength 0–100 mM NaCl or KCl). The Pfk B activity is tenfold lower than that of Pfk A but it is not allosterically inhibited by reaction products and physiologic levels of selected metabolites. In contrast, Pfk A is inhibited by increasing concentrations of reaction products (F16bP and ADP), as well as by glycolytic intermediate (PEP) and citrate. Our results also indicate that Pfk B can catalyze the reverse metabolic flux albeit with lower efficiency. Thus, Pfk B can serve as basic catalyst of glycolytic reaction under conditions inhibiting activity of main Pfk A enzyme. The detailed knowledge of Mtb enzymes participating in metabolic regulation may bring a new light to efficient antimycobacterial compounds development.

## 4. Materials and Methods

### 4.1. Cloning, Expression and Purification of Mtb Pfk A and Pfk B

The coding sequences of Mtb Pfk A (Rv3010c) and Pfk B (Rv2029c) were cloned into pET 15b and pET 20b vectors (Novagen, Madison, WI, USA), respectively. Pfk A was cloned in-frame with an N-terminal His-tag and Pfk B with a C-terminal His-tag. *E. coli* BL21 (DE3) cells containing the plasmid pGKJE8 for co-expression of chaperones (Chaperone Plasmid Set, Takara, Otsu, Japan) were used for expression of both isoenzymes. Cultivation was performed in LB medium supplemented with kanamycin (30 µg/mL) and chloramphenicol (20 µg/mL) for plasmid selection and with L-arabinose (1 mg/mL) and tetracycline (5 ng/mL) for induction of chaperone (dnaK, dnaJ, grpE, groEL, groES) expression at 30 °C. When the cell culture reached an OD_600_ of approximately 0.6, Pfk expression was induced by addition of isopropyl β-d-1-thiogalactopyranoside (IPTG) to a final concentration of 0.4 mM, and the cells were incubated at 20 °C overnight. The harvested cells were resuspended in 20 mM Tris-HCl, pH 7.4, containing 1 M NaCl and protease inhibitor cocktail (LaRoche, Basel, Switzerland), and frozen at −70 °C. The cell walls were lysed by multiple freeze-thaw cycles and addition of lysozyme (0.5 mg/mL of supernatant), disrupted by a single pass through an Avestin Emulsiflex C3 operating at 1100 bar, and sonicated three times for 1 min. The insoluble fraction was removed by centrifugation at 15,000 rpm for 30 min at 4 °C. The supernatant was loaded onto Talon^®^ resin (Stratagene, San Diego, CA, USA), and the column was washed with 20 mM Tris-HCl, pH 8, containing 300 mM NaCl, 5 mM β-mercaptoethanol, and 10 mM imidazole. Recombinant proteins were eluted with a gradient of 10–800 mM imidazole.

Fractions containing Pfk A were pooled, concentrated using an Amicon Ultra centrifugal filter unit (Millipore, Darmsdtadt, Germany), and loaded directly onto an FPLC HiLoad^TM^ 10/300 GL column (Superdex^TM^ 75 pg, GE Healthcare, Chicago, IL, USA) equilibrated with 20 mM Tris-HCl, pH 8, containing 500 mM NaCl and 5 mM β-mercaptoethanol. Pooled fractions containing Pfk B were dialyzed against 20 mM Tris-HCl, pH 8, 300 mM NaCl and loaded onto an FPLC HiTrap^TM^ IMAC FF column (GE Healthcare) previously charged with Ni^2+^ ions according to the manufacturer’s protocol and equilibrated in 20 mM Tris-HCl, pH 8, containing 300 mM NaCl and 10 mM imidazole. The protein was eluted from the column with a gradient of 10–400 mM imidazole. Fractions containing Pfk B were pooled and concentrated using an Amicon Ultra centrifugal filter (Millipore) and loaded onto an FPLC HiLoad^TM^ 10/300 GL (Superdex^TM^ 75) column equilibrated with 20 mM Tris-HCl, pH 8, containing 500 mM NaCl and 5 mM β-mercaptoethanol. The column effluents were monitored at 280 nm, and fractions were analyzed by SDS-PAGE and Western blot using mouse monoclonal anti-polyhistidine antibodies conjugated with horseradish peroxidase (Sigma Aldrich, St. Louis, MO, USA). The aliquots of purified enzymes were stored at −70 °C. Enzyme concentrations were determined by quantitative amino acid analysis.

### 4.2. Synthesis of Fructose-2,6-Bisphosphate (F26bP)

The compound was prepared by two-step procedure from F16bP by intramolecular cyclisation to F6P-1,2-cyclicP followed by alkaline hydrolysis. We substantially modified product purification and isolation of previously described method [20,50].

#### 4.2.1. Cyclization

F16bP tri Na^+^ salt (500 mg; 1.23 mmol) was dissolved in water (2.5 mL), passed through a column of Dowex 50 (H^+^ form, 20 × 1.5 cm) and eluted with water. Appropriate fractions were passed through a column of Dowex 50 (pyridinium^+^ form, 20 × 1.5 cm), eluted with water and appropriate fractions evaporated to the final volume 5 mL. Pyridine (40 mL) and triethylamine (0.5 mL) were added, followed by a solution of dicyclohexylcarbodiimide (DCC, 2.5 g) in pyridine (10 mL). The mixture was stirred at room temperature for 48 h. The reaction course was monitored by TLC on a silica gel plate in system propan-2-ol—25% NH_4_OH—water (11:7:2) with PMA (phosphomolybdenic acid) detection: R_F_ of F16bP = 0.05, R_F_ of F6P-1,2-cyclicP = 0.3. The cloudy solution was diluted with water (50 mL), the solid filtered off and the filtrate extracted with diethyl ether (3 × 150 mL). An aqueous phase was concentrated to a volume approx. 5 mL and applied onto a column of DEAE Sephadex A25 (10 × 2 cm) activated with 0.02 M TEAB. Elution was performed with water (250 mL) followed by a gradient of TEAB (0–0.4 M, 600 mL). Single fractions (50 mL each) were analyzed by MS and ^31^P NMR. F6P-1,2-cyclicP was eluted with 0.3 M TEAB and lyophilized to give 173 mg (22 %) of a white solid of its Et_3_NH^+^ salt. Mass spectra: ESI MS, *m/z*: 642.8 [2M − H]^−^ (10), 320.9 [M − H]^−^ (100). High resolution electrospray mass spectrum (HRMS (-ESI): For C_6_H_11_O_11_P_2_ (M − H)^−^ calculated: 320.97821; found: 320.97815. ^31^P NMR (400 MHz, D_2_O): 5.36 (P-6), 18.75 (1,2-cyclic P).

#### 4.2.2. Alkaline Hydrolysis

Et_3_NH^+^ salt of F6P-1,2-cyclicP (173 mg; 0.28 mmol) was dissolved in 1 M NaOH (25 mL) and stirred for 10 min at 36 °C. This way, a mixture of two fructose bisphosphates was formed: F16bP as the main product together with a small amount of F26bP. To remove undesired F16bP, a different stability of both isomers towards strongly alkaline conditions was utilized. The solution was diluted with water (100 mL) and heated for 30 min to 90 °C (temperature of reaction mixture inside the flask). F16bP was degraded to F6P and inorganic phosphate while F26bP remained intact. Reaction mixture was then rapidly cooled in ice bath to room temperature and carefully neutralized by addition of small portions of Dowex 50 (H^+^) under vigorous stirring to pH 8.5. Ion exchanger was filtered off and the filtrate passed through a column (10 × 1 cm) of DEAE Sephadex A25 activated with 0.02 M TEAB. After that, the column was eluted with a very slow gradient of TEAB (0.2–0.4 M, 600 mL). All fractions (50 mL each) were separately lyophilized and analyzed by MS and NMR spectra. The majority of isolated material was F6P eluted at the concentration 0.2 M TEAB. The desired product was eluted in the range 0.35–0.40 M TEAB and lyophilized to give 5.7 mg of amorphous solid of F26bP in the form of Et_3_NH^+^ salt. NMR data are in agreement with literature [51].

Mass spectra: ESI MS, *m/z*: 339.2 [M − H]^−^ (100). High resolution electrospray mass spectrum (HRMS, -ESI): For C_6_H_13_O_12_P_2_ (M − H)^−^ calculated: 338.98877; found: 338.98737.

### 4.3. Measurement of Pfk Glycolytic Activity

Glycolytic activities of Pfk A and B were determined by two spectrophotometric methods. For majority of experiments the coupled assay I with aldolase, triosephosphate isomerase and glycerol-3-phosphate dehydrogenase was used. In this assay, F16bP is converted to dihydroxyacetone phosphate and glyceraldehyde-3-phosphate, which is reduced to glycerol-3-phosphate and NADH. The decrease in absorbance at 340 nm corresponding to NADH oxidation to NAD^+^, indirectly corresponds to F16bP production. For detection of Pfk A substrate specificity and activity regulation by metabolites, the coupled assay system II with pyruvate kinase and lactate dehydrogenase was applied. In this assay, formation of NAD^+^ during reduction of pyruvate to lactate was monitored.

The reactions of system I were carried out in 50 mM Tris-HCl, pH 7.4, 10 mM MgCl_2_, 1 U/mL triosephosphate isomerase (rabbit muscle, Sigma-Aldrich, St. Louis, MO, USA), 1 U/mL α-d-glycerol-3-phosphate dehydrogenase (rabbit muscle, Sigma-Aldrich, USA), 1 U/mL aldolase (rabbit muscle, Sigma-Aldrich, USA), 0.3 mM NADH and 30 nM Pfk A or 300 nM Pfk B (buffer RXN) at 37 °C. Initial velocity studies were performed by varying one substrate concentration (0–12 mM NTP or 0–50 mM F6P) while keeping the concentration of the second substrate constant (1 mM ATP or F6P). The dependencies of the reaction velocities on the concentration of NTP (A) or F6P (B) were performed in following arrangement: (A) 190 µL aliquots of the reaction buffer (RXN) with 1 mM F6P were pipetted to corresponding microtiter wells containing 10 µL of NTP (0.2, 1, 2, 5, 10, 15, 20, 50, 100, 120, 140, 160, 180, 200, 210, 250 mM ATP, GTP or ITP). (B) 190 µL aliquots of the reaction buffer (RXN) with 1 mM ATP were pipetted to corresponding microtiter wells containing 10 µL of F6P (PfkA: 10, 20, 40, 80, 120, 160, 200, 400, 800 mM; PfkB: 0.2, 0.5, 1, 2, 5, 10, 20, 100, 200, 400, 800 mM).

The assumption that Pfk B can utilize pyrophosphates (PP_i_) was tested using similar assay but with PP_i_ instead of ATP. 190 µL aliquots of the reaction buffer RXN with 0.5 mM F6P were pipetted to corresponding microtiter wells containing 10 µL of PP_i_ (1, 2, 3, 4, 5, 6, 7, 8, 9, 10, 20, 30, 40, 50, 60, 70, 80, 90, 100 mM).

All measurements described here and following chapters were carried out in 96-well plates (NUNC-polysorp, Thermoscientific, Waltham, MA, USA) equilibrated at 37 °C and the absorbance was recorded with a Tecan^®^ Infinite M1000 plate reader (Tecan, Männedorf, Switzerland). All experiments were performed in triplicate.

### 4.4. Determination of Pfks Substrate Specificity

#### 4.4.1. Sugar Substrate Specificity of Pfk A

Due to the fact that Pfk A is efficiently inhibited by PEP used in the coupled enzyme assay II a standard assay had to be modified. A total of 190 µL of reaction mix (50 mM Tris-HCl, pH 7.5, 10 mM MgCl_2_) containing 1 mM ATP, 1 U/mL pyruvate kinase, 1 U/mL lactate dehydrogenase, 300 µM NADH, and 30 nM Pfk A (coupled enzyme assay II) was pipetted into individual microtiter wells with 10 µL of a particular sugar-x-phosphate (fructose-6-phosphate, fructose-1-phosphate, tagatose-6-phosphate, sedoheptulose-7-phosphate, glucose-6-phosphate, ribulose-5-phosphate) (0.25, 0.5, 1, 2.5, 5, 6.7, 10, 20, 40, 100 mM) incubated at 37 °C. In the absence of PEP, the coupled enzyme assay could not be initiated and single Pfk A reaction proceeded for 5 min. Then 10 µL 100 mM PEP was added in to each well to start the subsequent coupled enzyme assay. The determined reaction velocity was directly proportional to the concentration of the formed ADP in the Pfk reaction. This corresponds to the average reaction velocity attained in the time interval 5 min related to the slope of secant line.

#### 4.4.2. Sugar Substrate Specificity of Pfk B

A total of 190 µL of reaction mix (50 mM Tris-HCl, pH 7.5, 10 mM MgCl_2_) containing 1 mM ATP, 1 U/mL pyruvate kinase, 1 U/mL lactate dehydrogenase, 300 µM NADH, 1 mM PEP, and 300 nM Pfk B (coupled enzyme assay II) was pipetted into individual microtiter wells with 10 µL of a particular sugar-x-phosphate (0.25, 0.5, 1, 2.5, 5, 6.7, 10, 20, 40, 100 mM) incubated at 37 °C.

All measurements were carried out in 96-well plates (NUNC-polysorp, Thermoscientific) equilibrated at 37 °C, and the absorbance was recorded with a Tecan^®^ Infinite M1000 plate reader (Tecan, Männedorf, Switzerland). All experiments were performed in triplicate.

### 4.5. Measurement of Pfk Gluconeogenic Activity

The gluconeogenic activity of Pfks was determined spectrophotometrically by monitoring the increase in absorbance in coupled enzyme reactions with phosphoglucose isomerase and glucose-6-phosphate dehydrogenase. The reaction velocity is proportional to the increase in absorbance at 340 nm due to NADP^+^ reduction to NADPH, which corresponds to the rate of F6P production in the Pfk-associated reaction. The reaction was carried out in 50 mM Tris-HCl, pH 7.4, 10 mM MgCl_2_, containing 0.2 mM NADP^+^, 3.5 U/mL phosphoglucose isomerase (rabbit muscle, Sigma-Aldrich, St. Louis, MO, USA), 2.5 U/mL glucose-6-phosphate dehydrogenase (baker’s yeast, Sigma-Aldrich, St. Louis, MO, USA) and 200 nM Pfk B (reaction buffer RXN). Initial velocity studies were performed by varying one substrate concentration (ADP or F16bP) while keeping the concentration of the second substrate constant (5 mM F16bP, 1 mM ADP). The dependencies of the reaction velocities on the concentration of NDP (ADP, IDP, GDP) (A) or F16bP (B) were performed in following arrangement: (A) 190 µL aliquots of the reaction buffer (RXN) with 5 mM F16bP were pipetted to corresponding microtiter wells containing 10 µL of NDP (0.2, 1, 20, 40, 100, 150, 200, 300 mM). (B) 190 µL aliquots of the reaction buffer (RXN) with 1 mM ADP were pipetted to corresponding microtiter wells containing 10 µL of F16bP (2, 10, 20, 100, 200, 400, 800 mM). All measurements were carried out in 96-well plates (NUNC-polysorp, Thermoscientific) equilibrated at 37 °C, and the absorbance was recorded with a Tecan^®^ Infinite M1000 plate reader (Tecan, Männedorf, Switzerland). All experiments were performed in triplicate.

### 4.6. Dependence of Pfk A and Pfk B activities on Mg^2+^ Concentration

A total of 190 µL of reaction mix (50 mM Tris-HCl, pH 7.5) containing 1 mM ATP or F6P, 1 U/mL aldolase, 1 U/mL triosephosphate isomerase, 1 U/mL glycerol-3-phosphate dehydrogenase, 300 µM NADH, 2, 3, 4, 5, 6, 7, 8, 9, 10, 12, 14, 15, 17, 19 or 20 mM MgCl_2_ and 30 nM Pfk A was pipetted into individual microtiter plate wells with 10 µL of F6P or ATP (0.25, 0.5, 1, 2, 4, 8, 12, 16, 20, 24, 28, 32, 36 mM).

A total of 190 µL of reaction mix (50 mM Tris-HCl, pH 7.5) containing 1 mM ATP and F6P, 1 U/mL aldolase, 1 U/mL triosephosphate isomerase, 1 U/mL glycerol-3-phosphate dehydrogenase, 300 µM NADH, and 300 nM Pfk B was pipetted into individual microtiter plate wells with 10 µL of MgCl_2_ (0.25, 0.5, 1, 2, 4, 8, 12, 16, 20, 24, 28, 32, 36 mM).

All measurements were carried out in 96-well plates (NUNC-polysorp, Thermoscientific, Waltham, MA, USA) equilibrated at 37 °C, and the absorbance was recorded with a Tecan^®^ Infinite M1000 plate reader (Tecan, Männedorf, Switzerland).

### 4.7. Testing Metabolites as Allosteric Modulators and Feedback Inhibitors

A total of 190 µL of reaction mix (50 mM Tris-HCl, pH 7.5, 10 mM MgCl_2_) containing 1 mM F6P or ATP, 1 U/mL aldolase, 1 U/mL triosephosphate isomerase, 1 U/mL glycerol-3-phosphate dehydrogenase, 300 µM NADH, 0–10 mM tested compound (ADP, GDP, citrate, G6P, R5P, PEP) and 30 nM Pfk A or 300 nM Pfk B was pipetted into microtiter plate wells containing 10 µL of 0.25, 0.5, 1, 2, 4, 8, 12, 16, 20, 24, 28, and 32 mM F6P or ATP.

To determine effect of F16bP on Pfk A, the modified uncoupled enzyme assay (termed here as assay II) was used, since 1 mM PEP completely inhibits Pfk A. 190 µL of the starting reaction mix (50 mM Tris-HCl, pH 7.5, 10 mM MgCl_2_) with 1 mM F6P or ATP, 1 U/mL pyruvate kinase, 1 U/mL lactate dehydrogenase, 300 µM NADH, 0–10 mM tested compound (F16bP) and 30 nM Pfk A was pipetted into microtiter plate wells containing 10 µL 0.25, 0.5, 1, 2, 4, 8, 12, 16, 20, 24, 28, and 32 mM F6P or ATP. The Pfk A catalyzed reaction without PEP proceeded for 5 min and then it was stopped by addition of 1 mM PEP, and simultaneously the subsequent coupled enzyme system including pyruvate kinase and lactate dehydrogenase was initiated. The average reaction velocity of Pfk corresponded to the ADP concentration in time period (5 min).

Pfk B is not inhibited by PEP and therefore the direct assay was used: 190 µL of reaction mix (50 mM Tris-HCl, pH 7.5, 10 mM MgCl_2_) containing 1 mM F6P or ATP, 1 U/mL pyruvate kinase, 1 U/mL lactate dehydrogenase, 300 µM NADH, 1 mM PEP, 0–10 mM tested compound (F16bP) and 300 nM Pfk B was pipetted into microtiter plate wells containing 10 µL of 0.25, 0.5, 1, 2, 4, 8, 12, 16, 20, 24, 28, and 32 mM F6P or ATP.

To test the effect of F26bP on Pfk A and Pfk B activities, the following experimental trials were prepared:

(1) The effect of F26bP on Pfk A or Pfk B with fixed concentration of one substrate and varied concentration of the second substrate: 190 µL of reaction mix (50 mM Tris-HCl, pH 8.0, 10 mM MgCl_2_, 5 mM DTT) containing 1 mM F6P or ATP, 1 U/mL aldolase, 1 U/mL triosephosphate isomerase, 1 U/mL glycerol-3-phosphate dehydrogenase, 300 µM NADH, 0–100 µM F26bP and 30 nM Pfk A or 300 nM Pfk B was pipetted into microtiter plate wells containing 10 µL of 0.25, 0.5, 1, 2, 4, 8, 12, 16, 20, 24, 28, 32, 36, 40 mM F6P or 1, 2, 4, 8, 12, 16, 20, 24, 28, 32, 40, 50, 60, 70, 75, 80 mM ATP.
(2) The effect of F26bP on the inhibition of Pfk A by citrate or phosphoenolpyruvate: 190 µL of reaction mix (50 mM Tris-HCl, pH 8.0, 10 mM MgCl_2_, 5 mM DTT) containing 1 mM F6P, 1 U/mL aldolase, 1 U/mL triosephosphate isomerase, 1 U/mL glycerol-3-phosphate dehydrogenase, 300 µM NADH, 1 mM citrate or 1 µM PEP, 0 or 50 µM F26bP and 30 nM Pfk A or 300 nM Pfk B was pipetted into microtiter plate wells containing 10 µL of 1, 2, 4, 8, 12, 16, 20, 24, 28, 32 mM ATP.
(3) To test the effect of F26bP on the inhibition of Pfk A by an excess of ATP: 190 µL of reaction mix (50 mM Tris-HCl, pH 8.0, 10 mM MgCl_2_, 5 mM DTT) containing 1 mM F6P, 1 U/mL aldolase, 1 U/mL triosephosphate isomerase, 1 U/mL glycerol-3-phosphate dehydrogenase, 300 µM NADH, 0 or 50 µM F26bP and 30 nM Pfk A was pipetted into microtiter plate wells containing 10 µL of 0.2, 1, 2, 5, 10, 15, 20, 50, 100, 120, 140, 160, 180, 200 mM ATP.

All measurements were carried out in 96-well plates (NUNC-polysorp, Thermoscientific) equilibrated at 37 °C, and the absorbance was recorded with a Tecan^®^ Infinite M1000 plate reader (Tecan, Männedorf, Switzerland).

### 4.8. Enzyme Kinetics Analysis

Steady-state kinetic rates were determined by measuring the slope of the tangent of the reaction progress curve. The initial velocity curves were fitted to the Michaelis-Menten equation (for hyperbolic regression analysis):

(1) $$\frac{v}{V^{\max}}=\frac{1}{1+\frac{K_{M}}{\left[S\right]}}$$

and the Hill Equation (for sigmoidal regression analysis):

(2) $$\frac{v}{V^{\max}}=\frac{1}{1+\frac{K_{1/2}}{{\left[S\right]}^{n}}}$$

where *K*_1/2_ is the half-maximal concentration and *n* is the Hill coefficient.

The data for biphasic kinetics was fitted using the following equation for the superposition of two sigmoidal functions:

(3) $$v=V_{1}^{\max}-\frac{V_{1}^{\max}}{1+{\left(\frac{\left[S\right]}{K_{1}}\right)}^{n}}+V_{2}^{\max}-\frac{V_{2}^{\max}}{1+{\left(\frac{\left[S\right]}{K_{2}}\right)}^{m}}$$

where *V*_1_^max^ + *V*_2_^max^ is the maximal velocity corresponding to the second plateau, and *V*_1_^max^ is the velocity corresponding to the first (middle) plateau in the biphasic curve. *K*_1_ and *K*_2_ are two mid-point substrate concentrations. The first and second Hill coefficients are *m* and *n*.

Inhibition of Mtb Pfk A by substrate (F6P, ATP) was modelled using Haldane’s Equation:

(4) $$v=\frac{V^{\max}.[S]}{K_{1/2}+[S]+{\left[S\right]}^{2}/K_{i,S}}$$

where *K_i,S_* is the substrate inhibition constant.

Individual saturation curves in the presence of different concentrations of inhibiting compounds were fitted using Equation (2).

The kinetic data were fitted and analyzed using the non-linear, least-squares, curve-fitting programs of Sigma Plot™ (SYSTAT Software Inc. version 11.0, Chicago, IL, USA). Error bars are standard deviations from three independent experiments.

## Figures and Tables

**Table 1:** Kinetic characteristics of Pfk A and Pfk B in the glycolytic reaction.

Glycolytic Reaction					
Isoenzyme	Substrate	V_max_ (µmol/min/mg)	K_1/2_ (K_m_) (mM)	n_H_	V_max_/K_1/2_ (L/min/mg)
Pfk A	ATP	19 ± 0.5	1.0 ± 0.2 K_i,ATP_ = 1.5 ± 0.3	1.6 ± 0.1	0.019 ± 0.004
Pfk A	F6P	20 ± 1	0.4 ± 0.1 K_i,F6P_ = 13.2 ± 2.0	1.6 ± 0.2	0.05 ± 0.01
Pfk B	ATP	0.8 ± 0.1 * 2.1 ± 0.1 **	0.052 ± 0.005 * 9.2 ± 0.1 **	1	0.015 ± 0.002
Pfk B	F6P	1.2 ± 0.1	0.04 ± 0.01	1	0.030 ± 0.008

The corresponding values were calculated from the curves presented in Figure 1. (*, **) correspond to values calculated from the first or second plateaus, respectively, in the biphasic response curve for Pfk B.

**Table 2:** Kinetic characteristics of Pfk B in the gluconeogenic reaction.

Gluconeogenic Reaction			
Substrate	V_max_ (µmol/min/mg)	K_m_ (mM)	V_max_/K_m_ (L/min/mg)
F16bP	0.39 ± 0.05	17.5 ± 1.5	2.2 × 10^−5^ ± 3 × 10^−6^
ADP	3.6 ± 0.2	12.9 ± 3.0	1.4 × 10^−4^ ± 4.9 × 10^−5^
IDP	0.37 ± 0.02	0.27 ± 0.05	1.4 × 10^−3^ ± 2.6 × 10^−4^
GDP	0.32 ± 0.07	0.28 ± 0.03	1.1 × 10^−3^ ± 2.8 × 10^−4^

The corresponding values were calculated from the curves presented in Figure 2.

**Table 3:** The kinetics of Pfk A and Pfk B with F6P and T6P substrates.

	K_1/2_ (mM)	V_max_ (µmol/min/mg)	V_max_/K_1/2_ (L/min/mg)
PfkA (F6P)	0.30 ± 0.05	22 ± 3	0.073 ± 0.015
Pfk A (T6P)	0.43 ± 0.05	30 ± 4	0.070 ± 0.012
Pfk B (F6P)	0.040 ± 0.005	1.2 ± 0.1	0.030 ± 0.005
Pfk B (T6P)	0.090 ± 0.005	1.5 ± 0.2	0.017 ± 0.002

The corresponding values were calculated from curves presented in Figure 7 and Figure S7 using the Equation (1) (Material and Methods) and Equation (S2) (Supplementary Materials).

## Data Availability

All experimental data supporting findings of this study are available upon corresponding author request.

## Supplementary Materials

The following are available online at https://www.mdpi.com/1422-0067/22/3/1483/s1.
