# Supplementary material for: Phosphofructokinases A and B from Mycobacterium tuberculosis Display Different Catalytic Properties and Allosteric Regulation
Source: Int J Mol Sci. 2021 Feb 2;22(3):1483. doi: 10.3390/ijms22031483 (PMC7867265; doi:10.3390/ijms22031483)
Supplement: Supplementary file 1 [file ijms-22-01483-s001.pdf]

Supplementary Materials for

Phosphofructokinases A and B from *Mycobacterium tuberculosis* display different catalytic properties and allosteric regulation

Jan Snášel[1], Iva Machová[1$], Veronika Šolínová[1], Václav Kašička[1], Marcela Krečmerová[1], and Iva Pichová[1*]

[1]Institute of Organic Chemistry and Biochemistry of the Czech Academy of Sciences, Flemingovo nám. 2, 16610 Prague 6, Czech Republic

[$]Current address. Biomedical Centre, Faculty of Medicine in Pilsen, Charles University in Prague, Alej Svobody 1655/76, 323 00 Pilsen, Czech Republic

Table of contents:

1/ Inhibition and further kinetic analysis

2/ The effect of Mg^{2+} on Pfk A tested with F6P varied and ATP fixed and *vice versa*.

3/ Test of the effect of PEP, citrate, and GDP on coupled enzymes

4/ Testing the effect of F26bP on Pfk A and Pfk B activities

5/ Monitoring of sugar monophosphates phosphorylation by Pfk A and B using capillary electrophoresis

6/ Supplementary **Figures S1-S8**

7/ Supplementary **Table S1**

8/ Additional literature and reference list

1/ Inhibition and further kinetic analysis

Allosteric inhibition profiles were evaluated using double reciprocal and Dixon plots and the inhibition data were fitted to equations based on ***Scheme 1***:

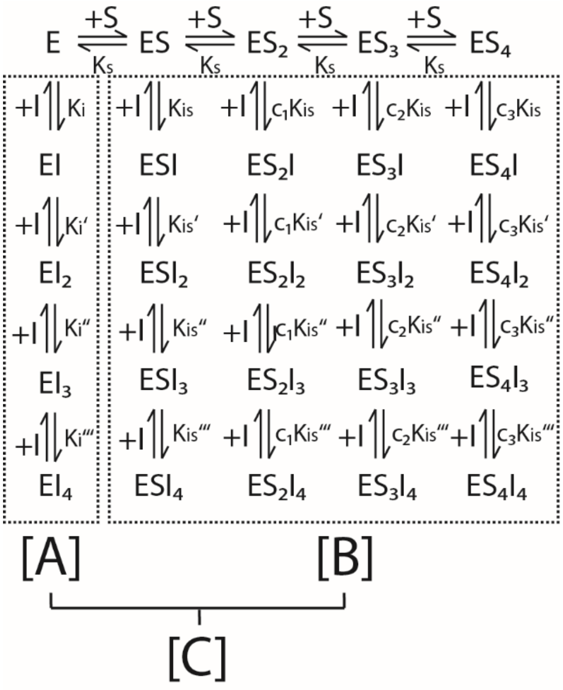

Scheme 1. *The multisite kinetic model for Pfk inhibition based on the homotetrameric enzyme.*

A generalized double reciprocal form of the equation for the fitting of the data is as follows:

$$1/v_i = a + b.[S]^{-n} \tag{S1}$$

With respect to individual cases of the allosteric inhibition the fitting parameters corresponded to

[**A**] in *Scheme 1*… $a=\frac{\gamma . K_s}{V^{\max}}$, $b=\frac{1}{V^{\max}}$ (if the inhibiting molecule binds only to free enzyme: E, …, E_n);

[**B**] in *Scheme 1*… $a=\frac{K_s}{V^{\max}}$, $b=\frac{\gamma}{V^{\max}}$ (if the inhibiting molecule binds to the enzyme-substrate complex: ES, …, E_mS_n);

[**C**] in *Scheme 1*… $a=\frac{\gamma . K_s}{V^{\max}}$, $b=\frac{\gamma}{V^{\max}}$ (if the inhibiting molecule binds to free enzyme or enzyme-substrate complex).

With respect to the multimeric state of the enzyme the inhibition parameter **γ** was as follows:

$$\gamma = 1+\frac{[I]}{K_{i(S)}}+\frac{[I]^2}{K'_{i(S)}}+\frac{[I]^3}{K''_{i(S)}}+\frac{[I]^4}{K'''_{i(S)}}. \quad \text{(S1a)}$$

$\mathbf{K_{i(S)}}$ is the dissociation constant of E-I_n (or ES-I_n) complexes. $\mathbf{K_s}$ is the half-saturation constant for the substrate S.

The effect of F16bP on Pfk A and the sugar-based substrate specificities of Pfk A were determined using kinetic analysis of progress curves. The equations used for the analysis were adapted from Orsi and Tipton, 1979 [1].

Using average reaction velocities Michaelis-Menten equation was rearranged to give (Henri equation):

$$\frac{[S]_0-[S]}{t}=V_{\max}-K_{1/2}.\frac{1}{t}.\ln\frac{[S]_0}{[S]} \quad \text{(S2)}$$

In which $\mathbf{[S]_0}$ is the initial concentration of the substrate and **[S]** is the concentration of the substrate in time **t**.

Evaluation and quantification of the allosteric effect (activation) caused by Mg^{2+} was determined according to methods described in [2], [3].

2/ The effect of Mg^{2+} on Pfk A tested with F6P varied and ATP fixed and *vice versa*. 190 µl reaction mix (50 mM Tris.HCl, pH 7.5) with 1 mM F6P or ATP, 1 U/ml aldolase, 1 U/ml triosephosphate isomerase, 1 U/ml α-D-glycerolphosphate dehydrogenase, 300 µM NADH and 0, 2, 3, 4, 5, 6, 7, 8, 9, 10, 12, 14, 16, 18 or 20 mM $MgCl_2$ was pipetted into microtiter plate wells containing 10 µl 0.25, 0.5, 1, 2, 4, 8, 12, 16, 20, 24, 28, and 32 mM F6P or ATP.

3/ Testing the effect of PEP, citrate, and GDP on coupled enzymes

190 µl reaction mix (50 mM Tris, pH 7.5, 10 mM $MgCl_2$) containing aldolase (1 U/ml), triosephosphate isomerase (1 U/ml), glycerol-3-phosphate dehydrogenase (1 U/ml), 1 mM F16bP, 300 µM NADH was pipetted into microtiter plate wells with 10 µl of 0, 20, 40, 60, 80, 100 µM PEP; 0, 10, 20, 30, 40 mM citrate; 0, 10, 20, 30, 40, 50, 60 mM GDP.

4/ Testing the effect of F26bP on Pfk A and Pfk B activities

To test the effect of F26bP on Pfk A and Pfk B activities the following reaction set ups were mixed: 190 µl reaction mix (50 mM Tris.HCl, pH 8.0, 10 mM $MgCl_2$, 5 mM DTT) containing 30 nM Pfk A or 300 nM Pfk B, 1 mM F6P, 1 U/ml triosephosphate isomerase, aldolase, glycerol-3-phosphate dehydrogenase, 300 µM NADH was added to the microtiter plate wells containing 10 µl of 2, 4, 8, 16, 30, 60, 120, 240, 500 µM, 1, 2 mM F2,6bP and 10 µl of 20 mM ATP.

4/ Monitoring of sugar monophosphates phosphorylation by Pfk A and B using capillary electrophoresis

Processing of different sugar phosphates by Pfks was monitored by capillary electrophoresis (CE). The typical reaction was performed in 50 mM Tris-HCl, pH 7.4, containing 5 mM $MgCl_2$, 5 mM DTT, ATP (0.1 mM for Pfk A and 1.0 mM for Pfk B), 1 mM F6P or 1 mM sugar phosphates. The following sugar phosphates were tested: fructose-1-phosphate (F1P), tagatose-6-phosphate (T6P), sedoheptulose-7-phosphate (S7P), glucose-6-phosphate (G6P) and ribulose-5-phosphate (Rib5P).

The reaction was started by addition of purified Pfk A or B to a final concentration of 20 nM or 200 nM, respectively, to the solution of sugar phosphates in reaction mixture and incubated at 30°C for 5, 10, 15, and 30 minutes. The reaction was stopped by boiling for 5 minutes. Analysis of the reaction mixture samples revealed that product/substrate (ADP/ATP) conversion rates were linearly dependent on time for up to 15 minutes. The enzyme activities achieved with individual sugar phosphates were related to the highest activity achievable with both enzymes and F6P. The data (relative activities in the presence of different sugar phosphates, i.e. normalized specific activities) presented in Table 3 were calculated from the CE analyses of two independent reaction mixtures.

Data were analyzed by measuring the quantity of ADP formed from ATP by CE. CE analyses were carried out with a P/ACE MDQ System (Beckman-Coulter, Fullerton, CA, USA), and data acquisition and evaluation were performed using the software P/ACE System MDQ, version Karat supplied by Beckman, and Clarity chromatographic and electrophoretic station (DataApex, Prague, CR). The CE apparatus was equipped with a UV-vis photodiode array spectrophotometric detector (190-600 nm) set at two detection wavelengths (206 and 254 nm). CE analyses were performed in an internally uncoated fused silica capillary,

total/effective length 40/30 cm, id/od 50/375 μm, with an outer polyimide coating (Polymicro Technologies, Phoenix, AZ, USA).

For each analysis, a new capillary was consecutively washed with 0.1 M NaOH, water, and the background electrolyte (BGE) (40 mM Tris, 40 mM Tricine, pH 8.1) with 2 bar pressure for 10 min each, and then conditioned in the BGE at a separation voltage of 25 kV for 20 min. Between analytical runs, the capillary was rinsed with the BGE with 2 bar pressure for 2 min. The capillary was thermostated by liquid coolant to 15°C.

For the analyses, 50 μl samples were taken from the reaction mixture and mixed with an equal volume of buffer (50 mM Tris-HCl, pH 7.4, 5 mM $MgCl_2$, 5 mM DTT). Subsequently, sample aliquots of 25-45 μl were mixed with an aqueous solution of the internal standard (1 or 5 mM sodium benzoate) to reach its final concentration of 0.1 mM ATP for Pfk A, and 1 mM ATP for Pfk B. The samples were injected hydrodynamically with 13.8 mbar pressure for 5-15 s. Baseline separation of ATP and ADP was achieved within 5-10 min at a separation voltage of 25.0 kV and electric current of 12.5-13.2 μA. The concentrations of ATP and ADP in the reaction mixtures were determined from their migration time corrected peak areas. The corrected peak areas were normalized to the corrected peak area of the internal standard (sodium benzoate) [4].

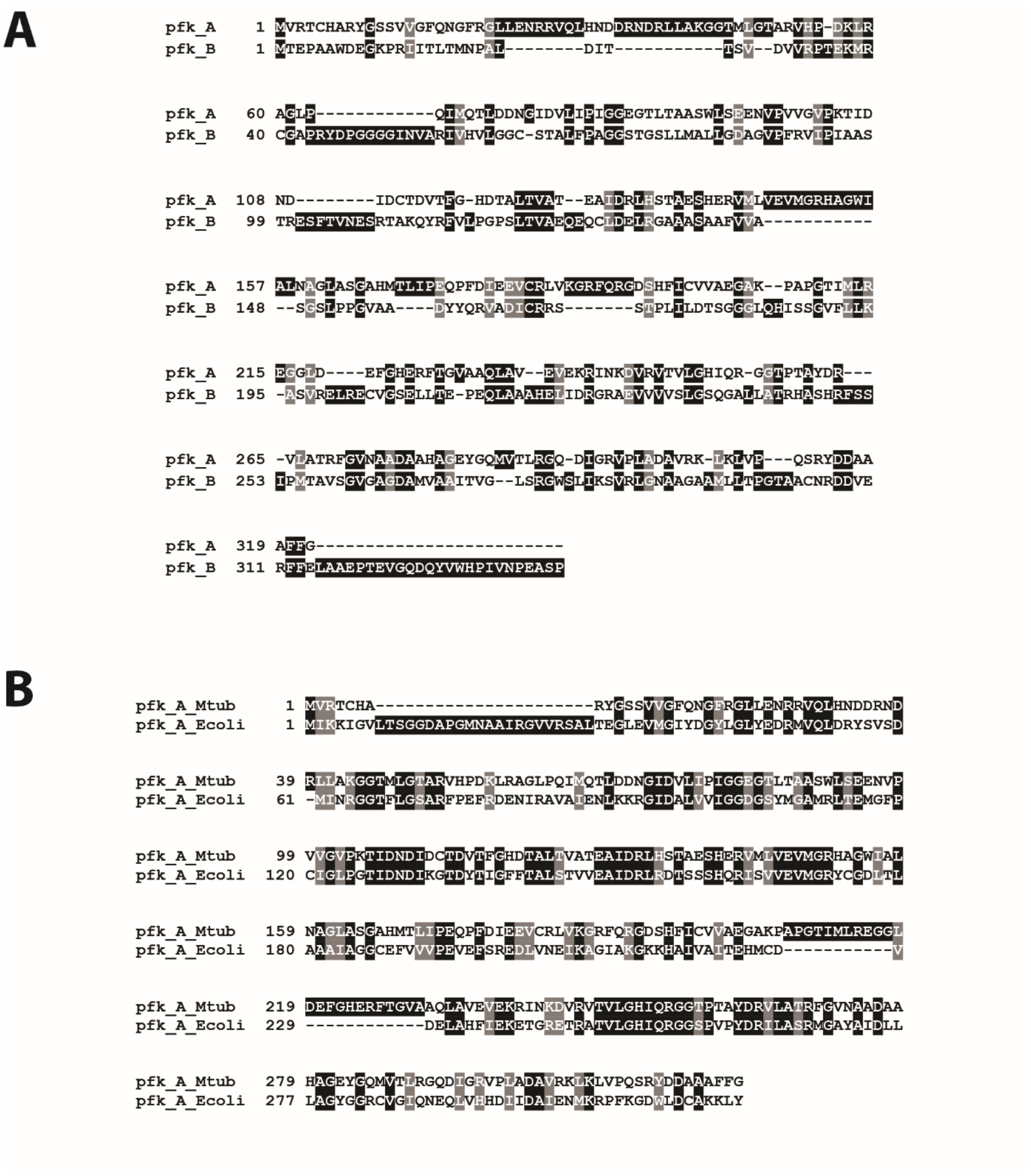

Figure S1. Amino acid sequence alignment of *Mtb* Pfk A and Pfk B (**A**) and *Mtb* Pfk A and *E. coli* Pfk A (**B**). The alignment was generated using the program CLUSTAL W in T-coffee (http://tcoffee.crg.cat/apps/tcoffee). Boxshade was used to generate publication-quality output (http://www.ch. embnet.org/software/BOX form.html). Gaps introduced for optimal alignment are marked by hyphens. Conserved regions are indicated as black boxes. Mtb, *Mycobacterium tuberculosis* (Pfk A:accession

number P9WID7; Pfk B:accession number P9WID3; UniProt); Ecoli, *Escherichia coli* (Pfk A:accession number D3QYB3; Pfk B:accession number D3QUR6).

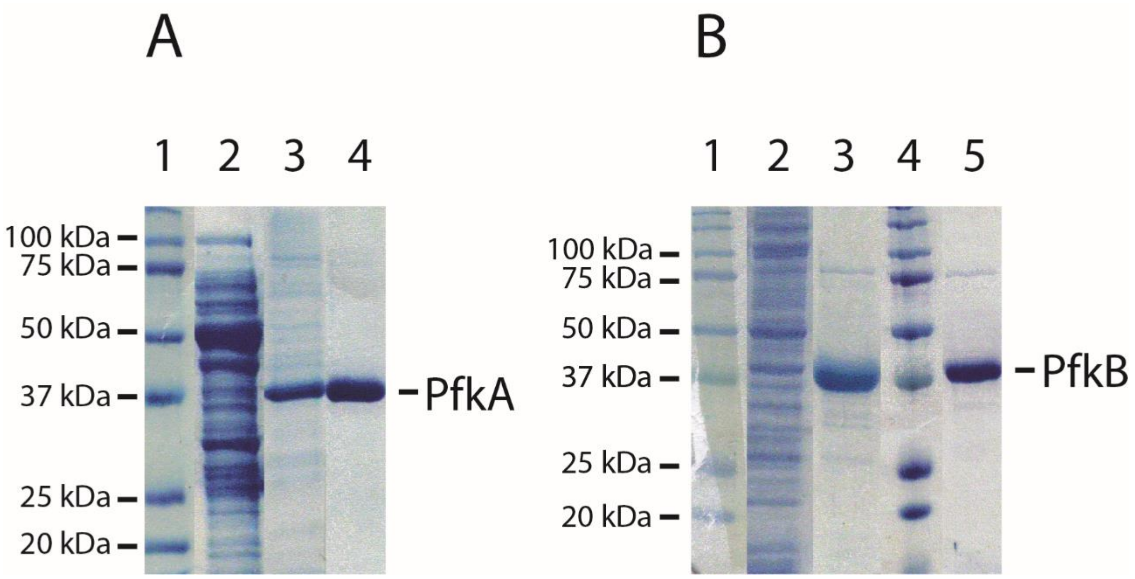

Figure S2. (A) SDS-PAGE analysis of Pfk A purification. Lane 1, molecular mass standards; lane 2, crude cell extract; lane 3, sample after chromatography on Talon resin; lane 4, sample after chromatography on Superdex 75. (**B**) **SDS-PAGE analysis of Pfk B purification.** Lane 1, molecular mass standards; lane 2, crude cell extract; lane 3, sample after chromatography on Talon resin; lane 4, molecular mass standards; lane 5, protein sample after chromatography on Superdex 75.

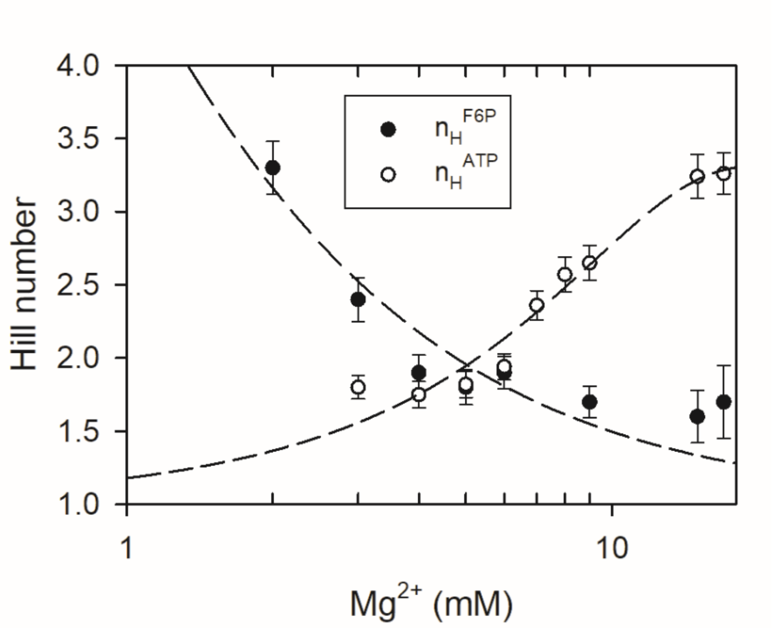

Figure S3. The effect of Mg^{2+} on Pfk A with F6P varied and ATP fixed at 1 mM (full circles) and with ATP varied and F6P fixed at 1 mM (open circles). The values of Hill numbers (n_H) were determined from fitting the steady state velocity data to eq. (2). The dashed line curves are drawn to show the trend of the data at each Mg^{2+} concentration.

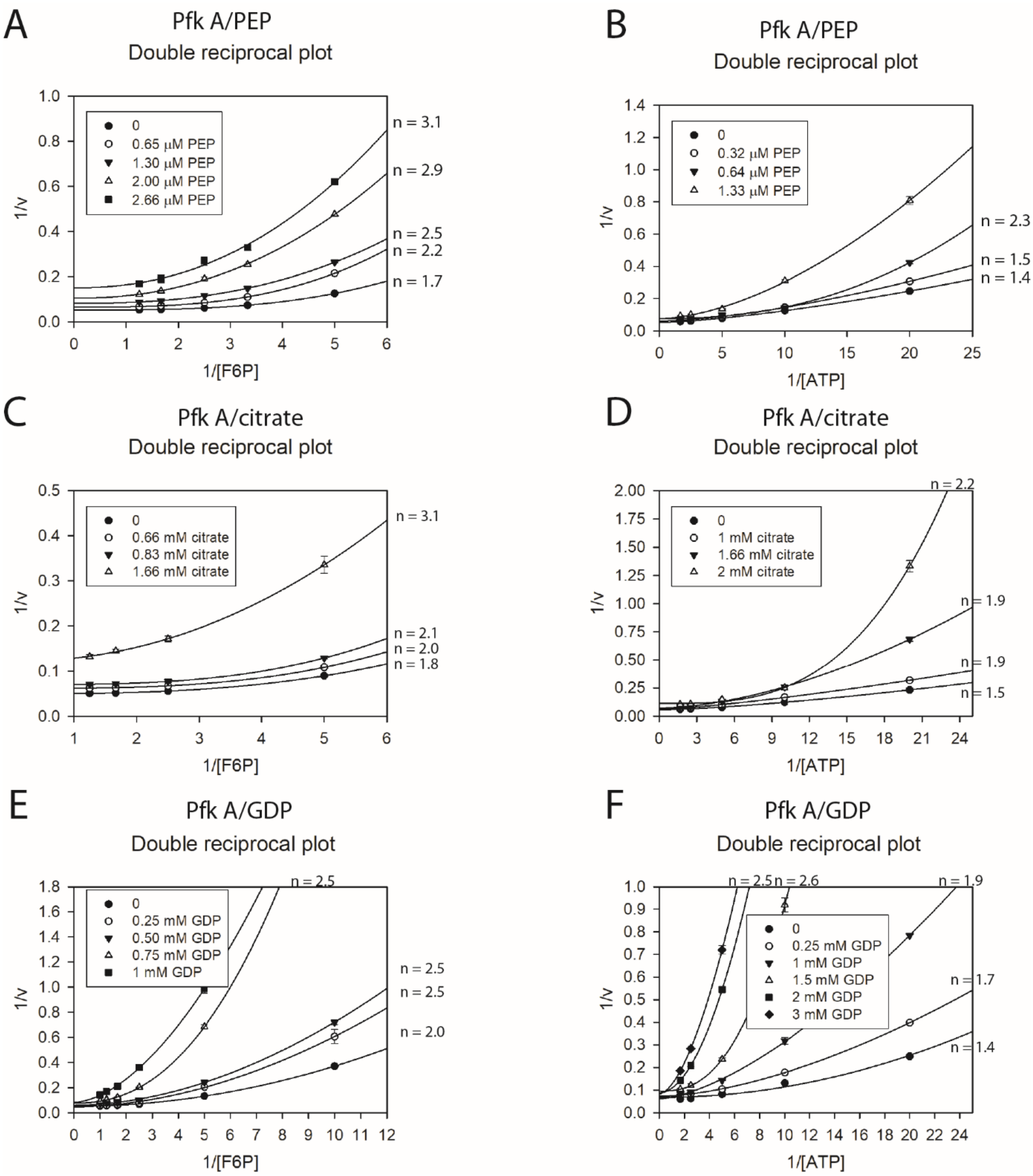

Figure S4. The inhibition of Pfk A by PEP, citrate and GDP. The double reciprocal plots for the inhibition of Pfk A by PEP, citrate and GDP at conditions with fixed concentration of ATP (1 mM) and varying concentrations of F6P (0-2 mM) (**A**), (**C**), (**E**) or at conditions with fixed concentration of F6P (1 mM) and varying concentrations of ATP (0-2 mM) (**B**), (**D**), (**F**). The data were fitted to eq. (S1). Corresponding Hill coefficients (n) are depicted above curves.

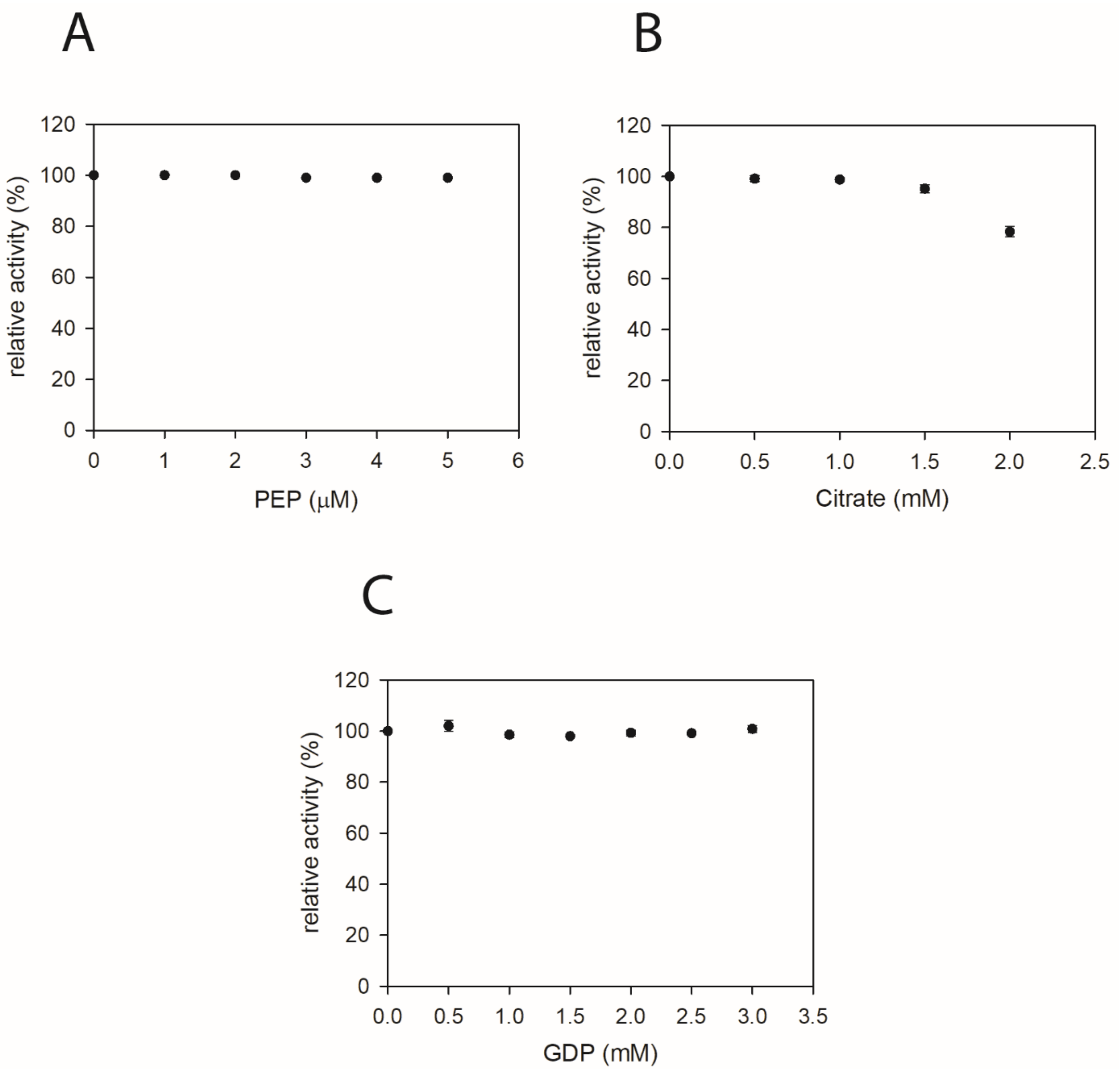

Figure S5. The effect of PEP (A), citrate (B), and GDP (C) on triosephosphate isomerase in the coupled enzyme assay. Coupled enzyme assay with 1 mM F16bP and triosephosphate isomerase, aldolase and glycerol-3-phosphate dehydrogenase (all 1 U) was run in the presence of 0-10 mM PEP, citrate and GDP. 100% corresponds to the activity achieved in the absence of any inhibiting compound.

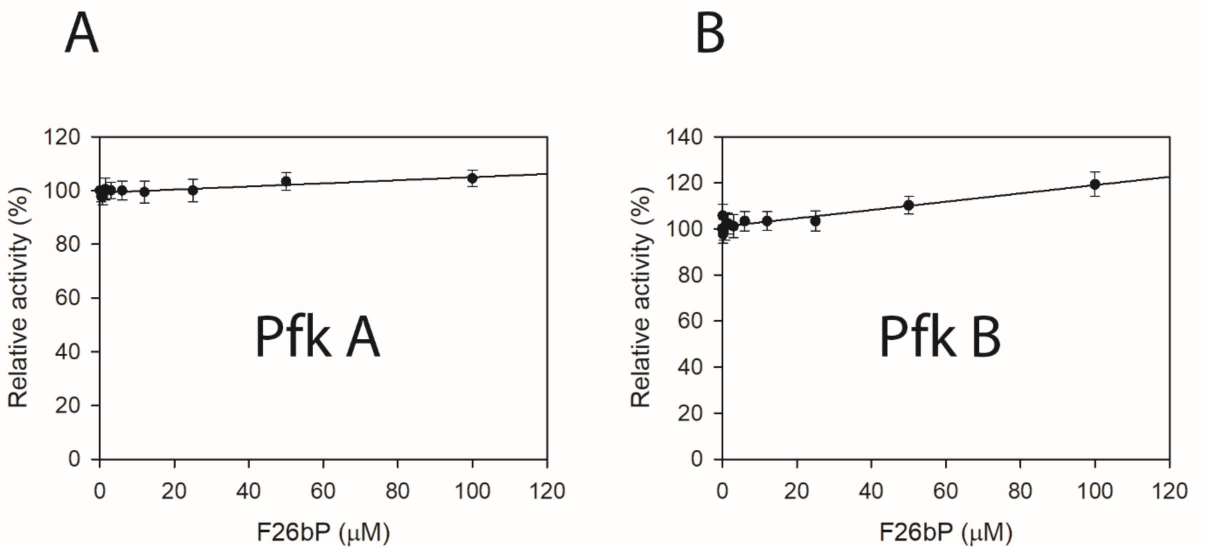

Figure S6. The activites of Pfk A and Pfk B in the presence of increasing concentrations of F26bP. 1 mM F6P and ATP was reacted in reaction mixtures containing increasing concentrations of F26bP (0-100 μM). For a better illustration the lines indicate the trend. 100% determines the activity achieved in the absence of F26bP.

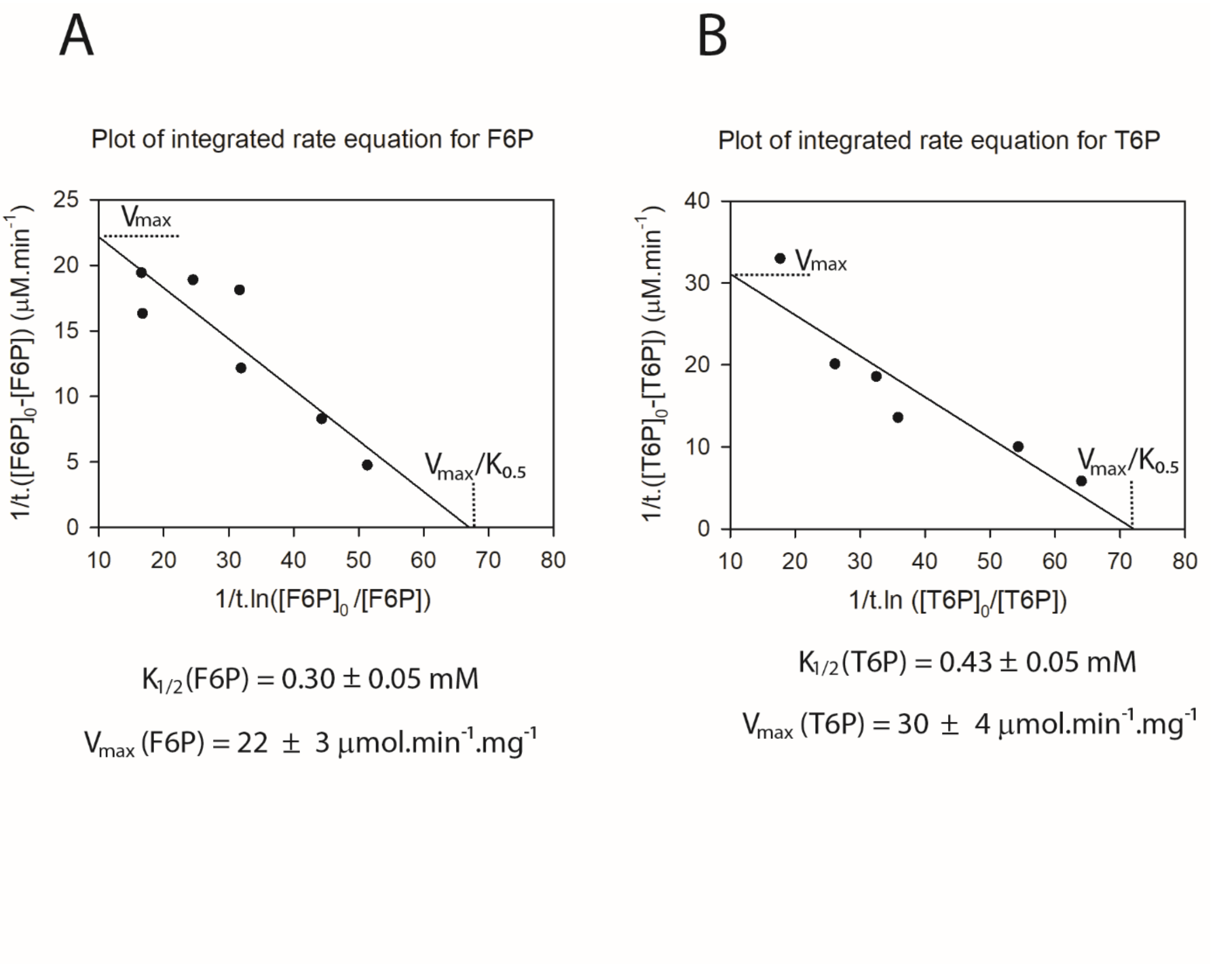

Figure S7. Plot of integrated rate equation (S2) for the Pfk A reaction with F6P (A) or T6P (B). The diagrams were constructed by plotting the data presented in Fig. 7A. The $[F6P]_0$, $[T6P]_0$ values represent starting substrate concentrations and [F6P], [T6P] values the substrate concentrations in 5 min. The form of the integrated rate equation (S2) is presented in the beginning of the Supplementary material section and was adapted from Orsi and Tipton [1].

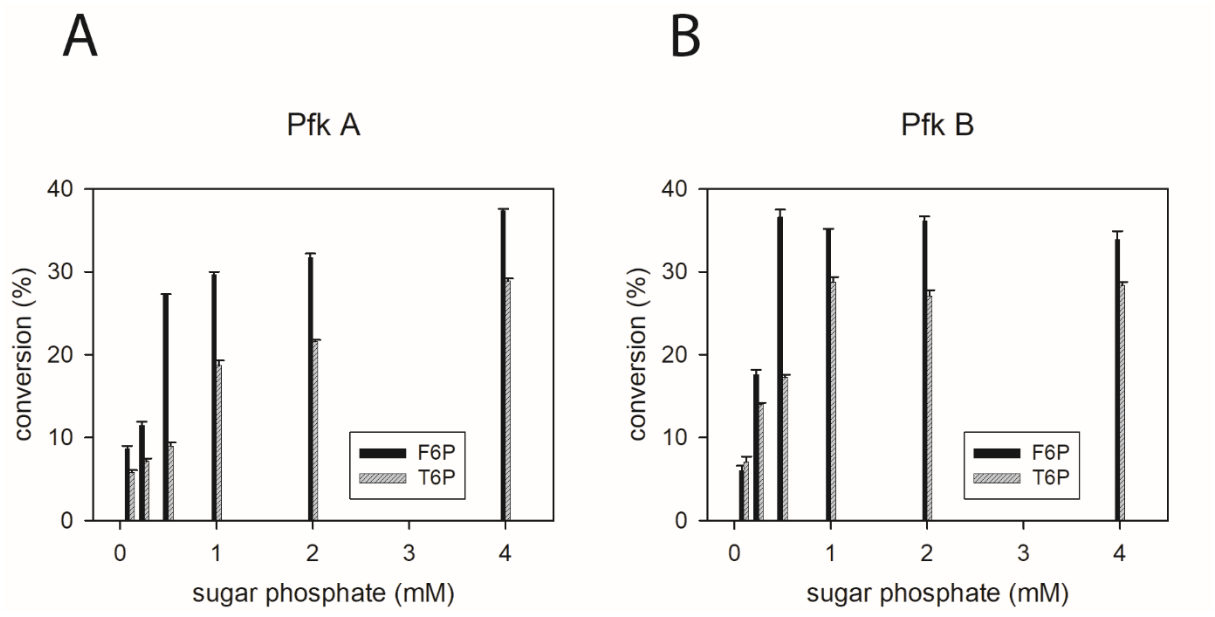

Figure S8. Use of different sugar phosphates by Pfk A (A) and Pfk B (B). 20 nM Pfk A or 200 nM Pfk B was mixed with 1 mM sugar phosphate, and 1 mM ATP as described in Material and Methods and reacted for 15 min at 30 °C. Conversion (%) is the amount of reactant (ATP) converted into product (ADP) per total amount or reactant as determined by capillary electrophoresis.

Table S1. Utilization of fructose-6-phosphate (F6P) and tagatose-6-phosphate (T6P) by Pfk A and Pfk B. 1 mM F6P or T6P was reacted with 1 mM ATP in the presence of 20 nM Pfk A or 200 nM Pfk B for 15 min at 30°C. The conversion (%) is the ratio of the formed ADP to unreacted ATP as determined by capillary electrophoresis. 100% corresponds to the highest conversion achievable.

	**F6P**	**T6P**
**Pfk A**	100%	63%
**Pfk B**	100%	82%

Supplementary material references:

[1] Orsi, B.A.; Tipton, K.F. Kinetic analysis of progress curves. *Methods Enzymol.* **1979**, *63*, 159-183.

[2] Mesecar, A.D.; Nowak, T. Metal-ion-mediated allosteric triggering of yeast pyruvate kinase. 1. A multidimensional kinetic linked-function analysis. *Biochemistry* **1997**, *36*, 6792-6802.

[3] Reinhart, G.D. Linked-function origins of cooperativity in a symmetrical dimer. *Biophys. Chem.* **1988**, *30*, 159-172.

[4] Šolínová, V.; Kašička, V.; Koval, D.; Barth, T.; Ciencialová, A.; Žáková, L. Analysis of synthetic derivatives of peptide hormones by capillary zone electrophoresis and micellar electrokinetic chromatography with ultraviolet-absorption and laser-induced fluorescence detection. *J. Chromatogr. B* **2004**, *808*, 75–82.
